# Expert-augmented machine learning for predicting extubation readiness in the pediatric intensive care unit

**DOI:** 10.1186/s12911-025-03070-z

**Published:** 2025-07-01

**Authors:** Jean Digitale, Deborah Franzon, Jin Ge, Charles McCulloch, Mark J. Pletcher, Efstathios D. Gennatas

**Affiliations:** 1 Department of Epidemiology and Biostatistics, University of California, San Francisco, San Francisco, CA USA; 2 Department of Pediatrics, Benioff Children’s Hospital, University of California, San Francisco, San Francisco, CA USA; 3 Division of Gastroenterology and Hepatology, Department of Medicine, University of California, San Francisco, CA USA

**Keywords:** Expert-augmented machine learning, Clinical prediction model, Machine learning, Interpretability

## Abstract

**Background:**

Determining extubation readiness in pediatric intensive care units (PICU) is challenging. We used expert-augmented machine learning (EAML), a method that combines machine learning with human expert knowledge, to predict successful extubation.

**Methods:**

We extracted electronic health record data from patients in two PICUs. Data from patients in one unit was split into 80% training and 20% test, while patients in the other served as an external test set. EAML begins by training RuleFit, which converts gradient-boosted trees into decision rules. Then, expert clinicians were asked to assess the relative probability of successful extubation of the subgroup defined by each rule compared with the entire sample. The rules were ranked in order of increasing chance of successful extubation according to (1) the RuleFit model and (2) clinician assessment, and differences between the two rankings were calculated. The initial RuleFit model was then regularized based on these differences, producing the EAML model.

**Results:**

The RuleFit model selected 46 rules; we surveyed 25 clinician experts to provide feedback on them. All clinicians worked in a PICU setting and were from multiple disciplines; over half (56%) had > 5 years of PICU experience. As expected, the added regularization slightly lowered performance of EAML compared with RuleFit in the internal test set, although the difference was not statistically significant (RuleFit AUC = 0.817 vs. best-performing EAML model AUC = 0.814, difference = 0.003, 95% CI of difference = -0.009, 0.003). EAML had superior performance in the external test set (RuleFit AUC = 0.791 vs. best-performing EAML model AUC = 0.799, difference = 0.007, 95% CI of difference = 0.002, 0.013).

**Conclusions:**

When creating a model to predict successful extubation in PICU patients, incorporating expert knowledge directly into the model construction process via EAML produced a model more generalizable to an external test set.

**Supplementary Information:**

The online version contains supplementary material available at 10.1186/s12911-025-03070-z.

## Background

Determining when a child in the pediatric intensive care unit (PICU) is ready to be successfully extubated is challenging and complex. Evidence-based protocols for liberation from mechanical ventilation have been shown to improve outcomes for adults compared with clinical judgment alone [1–5], but this has not been replicated in children [6, 7]. The lack of consensus on guidelines for extubation of critically ill children [8, 9] means that individual clinician judgment is the primary determinant of extubation decision-making in the PICU [10, 11]. The consequent variation in timing of extubation may be detrimental to subsets of patients because increased morbidity and mortality may arise from premature or delayed extubation [7, 12]. Successfully extubating children earlier would lower the risk of adverse events including ventilator-induced lung injury, ventilator-associated pneumonia, and delirium [6, 7]. This must be balanced with minimizing extubation failure, which results in longer PICU length of stay [13, 14], higher costs [11], and, in some studies, higher mortality rates [13, 15].

A reliable, convenient tool to predict extubation readiness for PICU patients would be beneficial to clinicians [11, 16]. Most clinical indices are poor predictors of extubation failure and have not been reliably implemented at the bedside [8, 12, 17]. The most widely used is the spontaneous breathing trial (SBT) [12], which involves setting the ventilator to minimal support to assess whether a patient is likely to be able to breathe independently if extubated [17]. This too has shown mixed results of effectiveness in children [6, 16, 17, 18, 19]. Furthermore, clinicians still commonly override the results of the SBT [20, 21]. In one study, for example, physicians decided to extubate only 66% of PICU patients who passed an SBT. Reasons for choosing not to extubate were primarily physician preference (26%) and excessive secretions (25%) [21]. Thus, the SBT does not fully capture all aspects that guide clinician choice about when to extubate critically ill children. Machine learning (ML) algorithms can incorporate many more inputs into predictions than a single test like the SBT and could be used to create a more complete picture of extubation readiness.

However, ML algorithms depend on good quality training data and, without any knowledge of true causal or biological contexts, can make mistakes that human clinicians would easily avoid [22]. In one classic example, a model for triage of patients with pneumonia [23] predicted that patients with asthma had a lower probability of death than those without. This prediction was based on a real pattern in the training data—patients with asthma were admitted directly to the intensive care unit because they were recognized by clinicians as having a higher risk of death [24]. Due to the effectiveness of this aggressive treatment, they had lower mortality than the general population. Yet, blindly basing treatment plans on the model’s predicted risk would result in less aggressive treatment for patients with asthma, leading to worse outcomes. Humans integrate their own subject matter knowledge and clinical experiences when making decisions at the bedside in a way that ML cannot.

A recently described approach combining ML and expert opinion, expert-augmented machine learning (EAML) capitalizes on the strengths of each [22]. First, an ML model creates an ensemble of decision rules, each of which defines subgroups of the population. Expert clinicians are asked to assess the relative probability of the outcome for the subgroup defined by each of these rules compared with the entire sample. The original ML model and expert assessment are then combined to create a final, hopefully more robust, model. Because the rules from the model are reviewed by human experts, human quality assurance can uncover hidden confounders and misleading patterns in the data, such as in the prior asthma example. Eliminating such spurious correlations may improve out-of-sample performance. Here, we apply EAML to the prediction of extubation readiness (successful extubation with no reintubation within 48 h) in the PICU.

## Methods

### Study population

Patients > 30 days and < 18 years old in PICUs at two children’s hospitals within an academic health system intubated between January 1, 2013 and March 31, 2023 were eligible for inclusion. The data for a patient was included if their intubation lasted > 24 h. Data from intubations lasting < 24 h were excluded as such patients were likely to be intubated for surgeries, procedures, or other indications and extubated quickly without complications. Patients intubated multiple times during the study period were considered for inclusion with each individual intubation event. Patients were excluded if they had a tracheostomy at the time of admission. Data from patients who died while intubated or died within 48 h of extubation were included, excluding the 24 h preceding extubation or death (whichever occurred first). Patients with congenital cardiac disease were excluded as extubation criteria in these patients may differ. PICU A is a 20-bed quaternary care unit in an academic regional referral children’s hospital, delivering care to children with high medical complexity and acuity, including transplantation. PICU B is a 23-bed community-based freestanding tertiary care children’s hospital and trauma center. Both PICUs use the same Epic-brand electronic health record (EHR) system. We received ethical approval from the University of California, San Francisco Institutional Review Board (study #17-23751).

### Data

EHR data were extracted from the institutional clinical data warehouse, which is updated daily from the real-time EHR. Variables included vital signs, ventilator settings, laboratory values, medications, neurological status, fluid balance, and other clinically relevant patient characteristics (Supplementary Table 1). For each intubation, predictions were made with these data every four hours, beginning at 12 h after intubation and ending approximately 12 h before extubation or other outcomes (endotracheal tube [ETT] change, transfer out of PICU, etc.; Supplementary Fig. 1). To facilitate this, the raw data were binned into 4-hour time windows (beginning at the time of intubation) containing the mean of numeric and binary variables and the mode of multi-level categorical variables (98 variables total). The first time window included in the model ended 12 h after intubation. For time-varying variables, we added the baseline value (first value available from time of intubation through the end of the first time window) and values from the two prior windows, resulting in 329 variables. More detail on feature inclusion is included in Supplementary Material 3. PICU A data was split 80% training, 20% test based on intubation date. PICU B data served as an external test set.

Our outcome was successful extubation (no reintubation within 48 h post-extubation). To create a binary variable, all outcomes other than successful extubation were grouped together: no extubation attempted, extubation failure, ETT change, patient transferred out of unit, etc. The outcome for each time window was extubation status 12 h later. This time-shifting was an attempt to preempt effects of clinician-enacted weaning actions. A prediction model is only useful if it gives clinicians additional insight rather than simply confirming what they already know. If clinicians are in the process of preparing to extubate a patient when the model recommends extubation, it is less useful than if it prompts them to prepare for extubation before they have imminently decided to do so. Thus, we assigned each time window’s outcome as the status 12 h after the end of the time window (see Supplementary Material 3).

### RuleFit

RuleFit begins by training gradient boosted trees [25]. It builds shallow trees that are converted to rules, which are a transformation of the variables to a Boolean matrix (i.e., True/False) [22]. For example, a tree may translate to a logical rule “age < 10 years & respiratory rate < 30”. All patients that match both conditions will have a value of “1” (defining a subgroup of patients); all others will have a value of “0”. Least absolute shrinkage and selection operator (LASSO) regression, which produces a sparse model that avoids overfitting, is then fit on the binary matrix to select a subset of rules. RuleFit offers high interpretability while maintaining high accuracy compared with state-of-the-art methods, such as gradient boosting [25]. We used the LightRuleFit implementation in the R package rtemis [26]. We determined the optimal hyperparameters (learning rate and number of leaves for the gradient boosting model and lambda for the LASSO model) via cross-validation in the training set (see Supplementary Material 3).

### Surveying experts

**Fig. 1:**
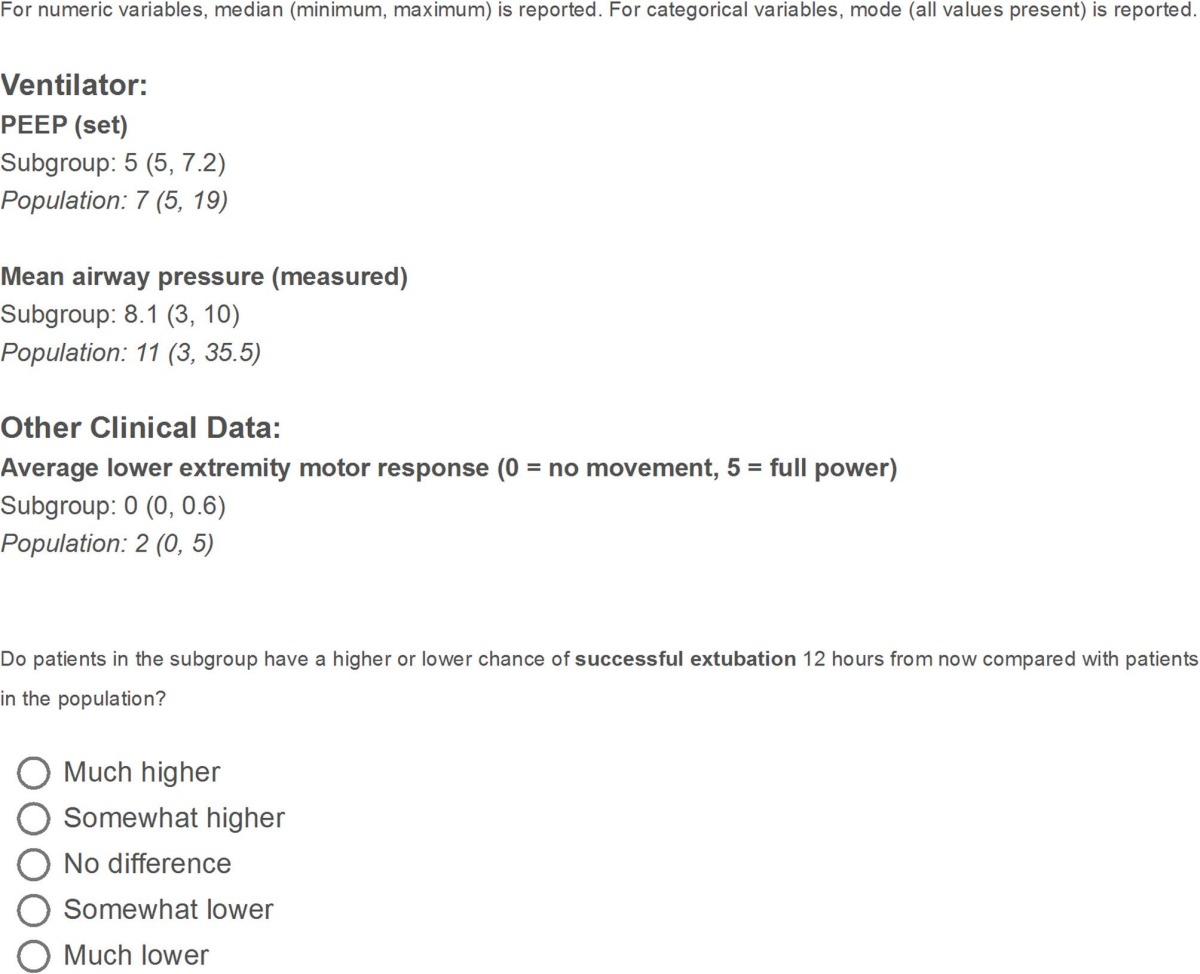
Example survey question

Expert clinicians were surveyed to assess the relative probability of successful extubation of the subgroup defined by each rule selected by LASSO compared with the full training sample in our data. The anonymous survey (Fig. 1 and Supplementary Material 3) was sent electronically to clinicians working in either one or both PICU locations. PICU nurses, respiratory therapists, pediatric critical care fellows, and attending physicians participated. For numeric variables, the survey displayed the median, minimum, and maximum of the subgroup and the full training sample. For categorical variables, the survey displayed the mode and all values present for both the subgroup and the full training sample. Respondents compared these values and used their clinical judgment to determine: “Do patients in the subgroup have a higher or lower chance of successful extubation 12 hours from now compared with patients in the population?” Answer options included: 1 = much higher, 2 = somewhat higher, 3 = no difference, 4 = somewhat lower, and 5 = much lower.

“Population” refers to the full training sample. Full survey available in Supplementary Methods.

### Expert-augmented machine learning

Survey responses were averaged (using numeric values indicated above) to determine which rules represented subgroups deemed more or less likely to be extubated successfully and the rules were ranked in order of increasing chance of successful extubation. The rules were also ranked in order of increasing empirical risk (probability of successful extubation in the subgroup defined by the rule in the training set) from the RuleFit model and the differences between the two rankings (delta rank) were calculated to represent the extent of disagreement between the human experts and model. To visually examine concordance, we created a scatterplot of the mean expert response vs. empirical risk. We described the rules that were highly discrepant, evaluated their face validity, and speculated about the reasons why there may have been disagreement.

To take into account the extent of inter-rater agreement between clinicians, the differences between model and expert rankings were divided by the standard deviation (SD) of the expert responses (delta rank/SD) [22]. This approach results in relatively higher penalization for rules where the experts agreed the most amongst themselves (smaller SD and larger resultant delta rank/SD). The delta rank/SD was plotted in a histogram to assess the distribution. The linear coefficients on the selected rules (from the initial RuleFit) were re-fit in the training set using ridge regression to incorporate clinician feedback in two different ways: (1) by using the absolute values as a penalty factor to allow differential shrinkage of the coefficients (soft EAML) and (2) by excluding the most discrepant rules as indicated by the most extreme values of delta rank/SD (hard EAML). Based on a histogram of delta rank/SD, models excluding 1, 3, 5, 7, 10, and 11 rules were compared. Performance (area under the receiver operating characteristic [ROC] curve [AUC]) of all EAML models in both test sets was reported. For each EAML model, differences between the AUC and the RuleFit AUC were calculated. Additionally, change scores were calculated by determining the difference between the test set AUC and the external test set AUC. To assess relative differences, the change score from the RuleFit model was subtracted from the change scores of all EAML models, resulting in a difference-in-differences measure. 95% confidence intervals (CIs) were constructed using bootstrapping with 2000 iterations per pairwise comparison. Analyses were run in R version 4.3.2 (R Core Team (2023)).

To assess differences in expert responses by role or years of experience, responses between groups for each rule were compared with histograms. As a sensitivity analysis, performance of EAML models was evaluated using only responses from attending physicians, the clinicians ultimately responsible for extubation decisions at the bedside.

## Results

### Sample characteristics

Our data included 1,436 intubations of 1,092 patients, resulting in 56,378 4-hour time windows. Sample description stratified by training, test, and external test sets is listed in Table 1. Median duration of intubation (days) varied slightly between groups: 4.3 (inter-quartile range [IQR] = 2.2, 8.2) in the training set, 4.8 (IQR = 2.2, 9.5) in the test set, and 3.5 (IQR = 1.7, 7.0) in the external test set. The most common primary diagnosis category was diseases of the respiratory system in the training set (25.5%), infectious diseases in the test set (30.9%), and injury, poisoning, and certain other consequences of external causes (e.g., trauma) in the external test set (30.8%). Descriptive statistics for variables at the time-window level are reported in Supplementary Table 2.

**Table 1:** Description of sample stratified by training, test, and external test set

Variable	Training	Test	External Test
**Patients**			
N	682	172	238
Sex = Female (%)	312 (45.7)	78 (45.3)	89 (37.4)
Race/Ethnicity (%)			
Asian	92 (14.2)	22 (13.3)	26 (11.2)
Black	55 (8.5)	9 (5.5)	38 (16.3)
Latinx	261 (40.4)	66 (40.0)	82 (35.2)
Other	57 (8.8)	28 (17.0)	55 (23.6)
White	181 (28.0)	40 (24.2)	32 (13.7)
**Intubations**			
N	940	233	263
Age at intubation (years) (median [IQR])	4.5 [1.0, 12.2]	4.9 [1.5, 12.2]	7.0 [1.7, 13.8]
Outcome (%)			
Extubation success	716 (76.2)	172 (73.8)	201 (76.4)
Extubation failure	88 (9.4)	23 (9.9)	8 (3.0)
Death	64 (6.8)	12 (5.2)	25 (9.5)
Tracheostomy	31 (3.3)	6 (2.6)	9 (3.4)
Transfer to another unit	16 (1.7)	13 (5.6)	17 (6.5)
ETT change	25 (2.7)	7 (3.0)	3 (1.1)
Duration of intubation (days) (median [IQR])	4.3 [2.2, 8.2]	4.8 [2.2, 9.5]	3.5 [1.7, 7.0]
Primary diagnosis (%)			
Diseases of the respiratory system	240 (25.5)	36 (15.5)	36 (13.7)
Certain infectious and parasitic diseases	120 (12.8)	72 (30.9)	61 (23.2)
Injury, poisoning and certain other consequences of external causes	84 (8.9)	14 (6.0)	81 (30.8)
Diseases of the nervous system	108 (11.5)	17 (7.3)	26 (9.9)
Neoplasms	77 (8.2)	20 (8.6)	15 (5.7)
Diseases of the circulatory system	75 (8.0)	23 (9.9)	12 (4.6)
Congenital malformations, deformations and chromosomal abnormalities	62 (6.6)	19 (8.2)	10 (3.8)
Diseases of the digestive system	56 (6.0)	7 (3.0)	3 (1.1)
Other	118 (12.6)	25 (10.7)	19 (7.2)

Patients intubated multiple times during the study period may have multiple intubation events included in the sample. The binary outcome of successful extubation for the model collapsed all other outcome categories. Here, extubation failure is defined as reintubation within 48 h, death is death before or within 48 h of extubation, and endotracheal tube (ETT) change is an extubation that was immediately and purposefully replaced by another ETT (e.g., to change the size). Primary diagnosis categories are based on Agency for Healthcare Research and Quality Clinical Classifications Software Refined (CCSR), v2024.1 [27]. Abbreviations: IQR = inter-quartile range

### Survey respondents

We surveyed clinician experts from multiple disciplines: 12 attending physicians, 7 fellows, 4 registered nurses, and 2 respiratory therapists. The largest group (32%) had > 10 years experience working in pediatric critical care; 24% had 6–10 years, 20% had 3–5 years, and 24% had < 3 years experience. The median time to complete the survey was 19.9 min.

### Agreement between model and experts

**Fig. 2:**
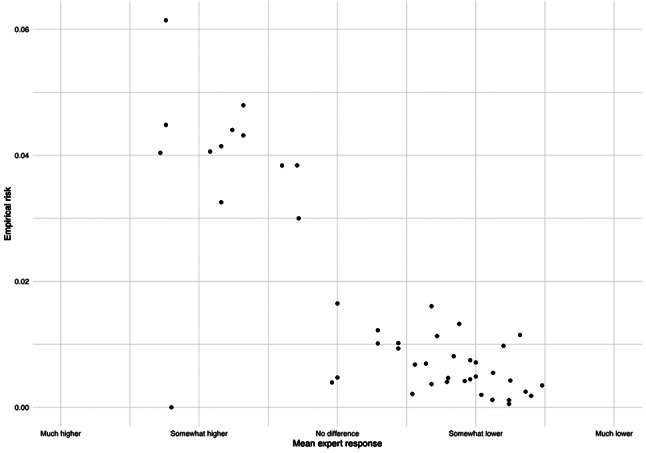
Mean expert response by empirical risk. Expert responses indicate whether patients in the subgroup have higher or lower chance of successful extubation 12 h from now, compared to patients in the full training sample. Empirical risk represents the probability of successful extubation in the training set within the subgroup defined by the rule

RuleFit selected 46 rules out of 96 initial gradient boosting rules. A scatterplot of mean expert response vs. empirical risk (probability of successful extubation) is displayed in Fig. 2. Overall, the model is concordant with expert consensus—rules with subgroups rated more likely to be extubated successfully by experts generally had higher empirical risk according to the model (R-squared = 0.66).

### Disagreement between model and experts

**Fig. 3:**
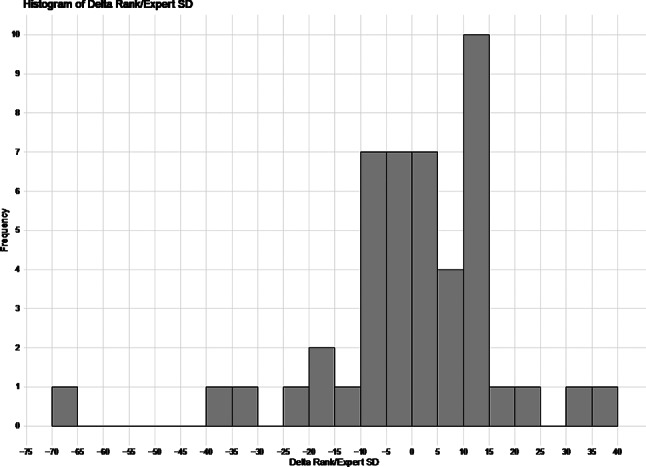
Histogram of delta rank/SD. Negative values of delta rank/SD indicate that model rated the subgroup defined by the rule less likely to be extubated successfully than the experts did. Positive values of delta rank/SD indicate the model rated the subgroup more likely to be extubated successfully than the experts did

A histogram of delta rank/SD is shown in Fig. 3. Negative values of delta rank/SD indicate that model rated the subgroup defined by the rule less likely to be extubated successfully than the experts did. Positive values of delta rank/SD indicate the model rated the subgroup more likely to be extubated successfully than the experts did. There is one outlier (Rule 45) with an extreme negative value of delta rank/SD and the rest of the distribution is reasonably symmetrical. This outlier had an empirical risk of exactly 0 (no chance of successful extubation according to the model), leading to the lowest model rank (1); however, it had one of the highest expert ranks (43). The expert responses for this rule averaged between much higher (1) and somewhat higher (2) chance of successful extubation (mean = 1.75) for the subgroup defined by the rule compared with the full training sample. This rule’s subgroup excluded patients charted as ineligible for an SBT 8–12 h ago, was slightly less sedated (higher average State Behavioral Scale score), and had only been intubated 12 h (much lower than the average duration of intubation). Empirical risk in the test set and external test sets were much higher (0.2 and 0.4, respectively) than in the training set, suggesting differences in populations in the outcome conditional on these parameters.

The most discrepant rules, accounting for SD, are displayed in Table 2. Of the top 11 most discrepant rules, 6 had negative and 5 had positive delta rank/SD. Data for all rules is shown in Supplementary Table 3.

**Table 2:** Rules that are most discrepant between model and experts based on delta rank divided by standard deviation (SD) of expert responses

Model rank	Expert rank	Expert SD	Delta Rank/ SD	Variable	Subgroup	Full training sample
1	43	0.65	-65.1	Spontaneous breathing trial: 8–12 h ago	Not assessed (Not assessed, Eligible but inconclusive, Failed, Passed)	Not assessed (Not assessed, Ineligible, Eligible but inconclusive, Failed, Passed)
				Hours intubated	12 (12, 12)	132 (12, 3072)
				State behavioral scale	-0.3 (-1.3, 2)	-1 (-3, 2)
30	4	0.69	37.7	Mean airway pressure (measured): 4–8 h ago	9 (3, 11.7)	11 (3, 35.5)
				Glasgow coma scale score: 8–12 h ago	4 (3, 6.7)	8 (3, 15)
17	32	0.41	-36.7	ETT placed day of surgery	No (No)	No (No, Yes)
				Respiratory rate (set)	16 (2, 20)	20 (0, 60)
				PEEP (set)	8 (6.7, 19)	7 (5, 19)
7	26	0.59	-32.3	Spontaneous breathing trial	Not assessed (Not assessed, Eligible but inconclusive, Failed, Passed)	Not assessed (Not assessed, Ineligible, Eligible but inconclusive, Failed, Passed)
				PEEP (set): 8–12 h ago	6 (5, 9)	7 (5, 19)
				Average lower extremity motor response (0 = no movement, 5 = full power)	0 (0, 0.4)	2 (0, 5)
22	13	0.29	31.2	FiO2: 4–8 h ago	60 (48.1, 100)	40 (21, 100)
26	8	0.82	22.0	Hours intubated	612 (336, 3068)	132 (12, 3072)
				PEEP (set): 4–8 h ago	6 (5, 9)	7 (5, 19)
11	33	1.06	-20.8	PEEP (set)	5 (5, 7.2)	7 (5, 19)
				Mean airway pressure (measured)	8.1 (3, 10)	11 (3, 35.5)
				Average lower extremity motor response (0 = no movement, 5 = full power)	0 (0, 0.6)	2 (0, 5)
4	10	0.33	-18.1	PEEP (set): 8–12 h ago	10 (9.2, 19)	7 (5, 19)
32	17	0.83	18.0	Hours intubated	412 (212, 3068)	132 (12, 3072)
				PEEP (set): 4–8 h ago	6 (5, 9)	7 (5, 19)
10	22	0.75	-16.0	Mean airway pressure (measured)	14 (11.5, 35.5)	11 (3, 35.5)
14	5	0.61	14.8	Pulse percentile (age-adjusted)	39.7 (0.1, 69.5)	69.6 (0, 100)
				Glasgow coma scale score: 4–8 h ago	3.8 (3, 7)	8 (3, 15)
				BMI z-score/Weight-for-length z-score: Baseline	-1.2 (-11.2, -0.1)	0.5 (-11.2, 7.6)

The rules were ranked in order of increasing probability of successful extubation in the subgroup defined by the rule (1 = lowest chance of successful extubation, 46 = highest chance of successful extubation). Negative values of delta rank/SD indicate that model rated the subgroup defined by the rule less likely to be extubated successfully than the experts did. Positive values of delta rank/SD indicate the model rated the subgroup more likely to be extubated successfully than the experts did. The table is sorted in order of the magnitude of delta rank/SD. Thus, in the EAML model in which the three most discrepant rules based on delta rank/SD were excluded, the first three rules listed in the table were removed. The subgroup and full training sample values listed are median (minimum, maximum) for numeric variables and mode (all values present in group) for categorical variables. Hours intubated was calculated as the number of hours between intubation and the end of the current time window. Abbreviations: BMI = body mass index; ETT = endotracheal tube; FiO2 = Fraction of inspired oxygen; PEEP = positive-end expiratory pressure

### RuleFit and EAML performance

**Fig. 4:**
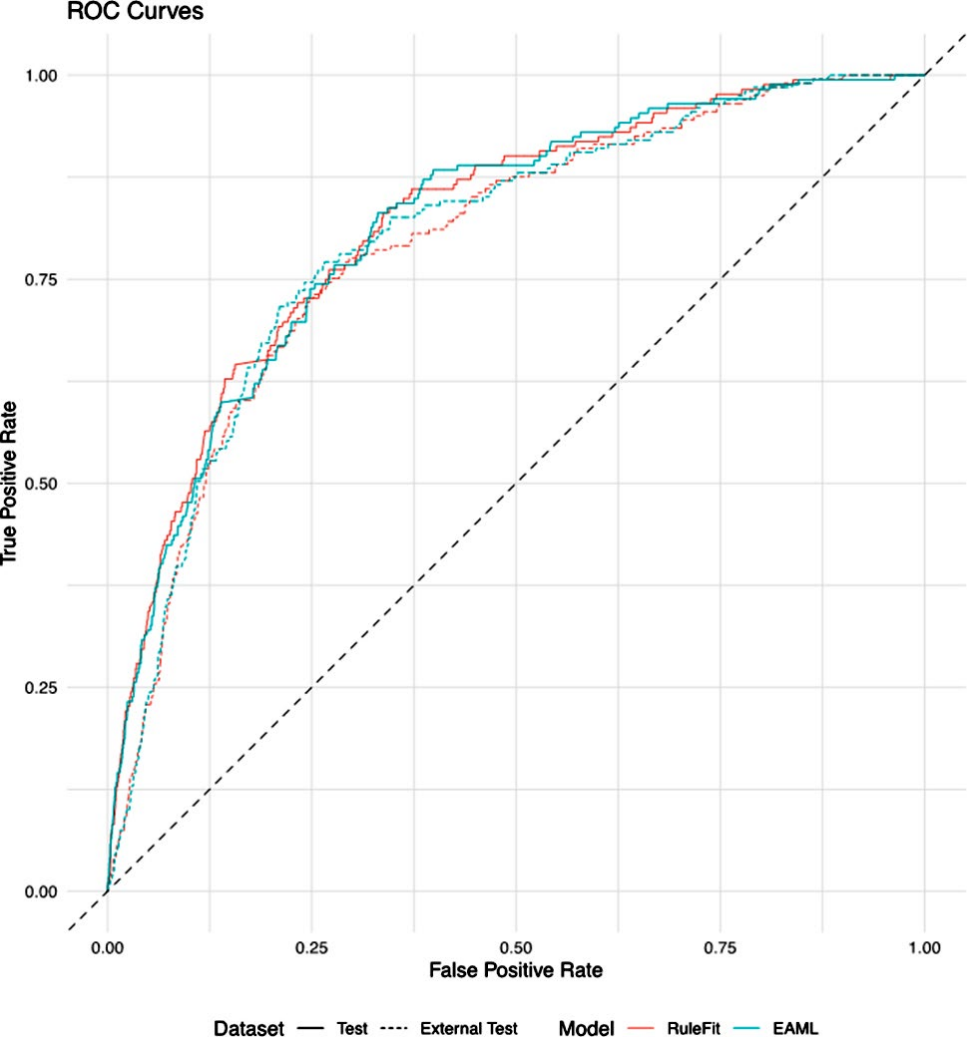
Receiver operator characteristic curves by test set. EAML model displayed has three rules excluded (highest EAML area under the curve in both test sets)

Reports model performance pre- (RuleFit) and post-incorporation of expert knowledge (EAML). The EAML model in both test sets with the highest AUC was the model excluding the three most discrepant rules. As expected, EAML performed worse in the internal test set (EAML: 3 rules excluded AUC = 0.814) compared with rulefit (AUC = 0.817) because rulefit had already minimized the cross-validated loss in the training sample and the internal test set shared the same correlation structure between the covariates and outcome. In the external test set, EAML (EAML: 3 rules excluded AUC = 0.799) performed better than rulefit (AUC = 0.791; difference = 0.007 [95% CI = 0.002, 0.013]). EAML with one rule excluded and five rules excluded also had AUCs that were statistically significantly larger than that of rulefit. The performance drop from the test set to the external test set was smaller for EAML models (AUC decrease: 0.013 to 0.017) compared to rulefit (AUC decrease: 0.026), indicating better generalizability of the EAML models. Figure 4 displays the ROC curves. Other model performance metrics are reported in Supplementary Table 4.

**Table 3:** Area under the receiver operating characteristic curve (AUC) pre- and post-incorporation of expert knowledge in the test and external test sets

Model	Test		External Test		Difference in AUC between External Test and Test	Difference in AUC between External Test and Test compared with RuleFit (95% CI)
	AUC	Difference in AUC from RuleFit (95%CI)	AUC	Difference in AUC from RuleFit (95%CI)		
RuleFit	0.8171	Ref	0.7912	Ref	-0.026	Ref
EAML: Soft	0.8123	-0.005 (-0.013, 0.003)	0.7957	0.005 (-0.002, 0.011)	-0.017	0.009 (-0.001, 0.02)
EAML: 1 rule excluded	0.8142	-0.003 (-0.008, 0.002)	0.7970	0.006 (0.001, 0.011)	-0.017	0.009 (0.001, 0.016)
EAML: 3 rules excluded	0.8144	-0.003 (-0.009, 0.003)	0.7986	0.007 (0.002, 0.013)	-0.016	0.01 (0.001, 0.019)
EAML: 5 rules excluded	0.8130	-0.004 (-0.011, 0.003)	0.7971	0.006 (0.0004, 0.012)	-0.016	0.01 (0.001, 0.019)
EAML: 7 rules excluded	0.8096	-0.008 (-0.015, -0.001)	0.7967	0.006 (-0.0004, 0.012)	-0.013	0.013 (0.004, 0.022)
EAML: 10 rules excluded	0.8081	-0.009 (-0.016, -0.002)	0.7945	0.003 (-0.003, 0.01)	-0.014	0.012 (0.003, 0.022)
EAML: 11 rules excluded	0.8084	-0.009 (-0.016, -0.002)	0.7956	0.004 (-0.002, 0.011)	-0.013	0.013 (0.004, 0.022)

RuleFit does not incorporate expert knowledge; all expert-augmented machine learning (EAML) models do. Confidence intervals (CIs) for differences were constructed using bootstrapping with 2000 iterations per pairwise comparison

### Differences in responses by role and years experience

There were differences in expert responses by role and years of experience (Supplementary Fig. 2), which resulted in slightly different rankings among subgroups. When comparing the 11 most discrepant rules (based on delta rank/SD) for attending physicians vs. the full sample, 8 rules overlapped (Supplementary Table 5). Performance metrics for EAML models were similar for the attending physician-only models, with 95% confidence intervals for the difference between EAML and RuleFit AUC excluding zero for the same three models in the external test set (Supplementary Table 6).

## Discussion

We used a novel method combining ML and human expert knowledge, EAML, to predict extubation readiness in PICU patients. The signal that ML models are able to detect is limited by the finite set of cases and features present in the data and they often struggle to maintain performance when applied to new cases. In contrast, experts have broader domain knowledge on which to draw. EAML aims to efficiently integrate expert-led quality control. It has the potential to identify miscoded variables and hidden confounders and can improve out-of-sample performance [22]. We found that incorporating expert knowledge into an ML model made it more generalizable in our experiment, improving performance in the external test set. Note that RuleFit was already fit to minimize cross-validated loss in the training sample and it has minimal overfitting because of the LASSO step. Thus, as expected, EAML did not improve performance in the internal test set (which shared a distribution with the training data). Further regularization by definition increases training error, so expert input could not improve model fit. In the external test set, however, EAML models (including human input) had higher AUCs than the original RuleFit model (without human input). Differences were small and, in this case, unlikely to be clinically meaningful, presumably because the external test set patient population was not vastly different from the training set patient population.

EAML shows promise to build models that maintain robust performance across distinct populations and sites. The change in performance between the test and external test sets was smaller for EAML models than for the RuleFit model. Pediatric critical care units generally have fewer mechanically ventilated patients than corresponding adult units [28] and research in the pediatric population usually has smaller sample sizes [29], making it difficult to train models separately for individual sites. Given this, EAML could be particularly beneficial in pediatrics to build models on training sets at larger hospitals and refine them with expert input for use at other centers.

Expert input revealed multiple rules that appeared to be spurious. For example, experts rated similarly two rules (Rules 14 and 26) for which the subgroup had a low median Glasgow Coma Scale [GCS] compared with the full training sample. However, the model ranking was highly discrepant for Rule 14 (second largest magnitude delta rank/SD), but less so for Rule 26 (17th largest). This suggests that there may be differences in the subgroups between the two rules that caused the model ranking to be discrepant for Rule 14. Additionally, with Rule 40 (third largest magnitude delta/rank SD), experts rated chance of successful extubation as no different from the full training sample with a relatively small SD, meaning they did not consider the rule to be predictive either way. Rules that are based on artifact are unlikely to generalize well to other samples because they may be overfit to the training data or based on non-causal correlations that are not present in external data. Expert input can aid in identifying such rules and mitigate this problem.

We treated expert consensus as the gold standard, but it is difficult to tell when that standard may be imperfect. Experts are human and can err. Our model weights expert assessment more heavily when there is agreement among them. Yet, if experts share a common bias, then their input is not a useful addition to the model. The effect of some variables may be inherently difficult to interpret or overweighted by experts. The subgroup for the most discrepant rule (Rule 45) had a very low median number of hours intubated in the subgroup (calculated as the number of hours between intubation and the end of the current time window), which may have been confusing to interpret. For example, a patient deemed clinically ready to extubate at the 12-hour mark may be expected to lack lung pathology and, therefore, have a higher chance of extubation success than another patient intubated much longer and deemed clinically ready to extubate. This 12-hour time anchor may have led experts astray, as it denoted simply that a patient was currently intubated at that time, not that clinicians attempted extubation. As evidenced by the median intubation duration of 4.1 days, most patients were not ready to extubate at that point. Additionally, two rules where the subgroups had much longer average intubation times than the full training sample (Rules 19 and 22) had large delta rank/SD (ninth and sixth largest, respectively). The number of hours intubated may be intrinsically difficult to extrapolate to extubation readiness without more information. Eventually, the vast majority of patients are successfully extubated, and longer intubation times do not always translate into higher likelihood of failure. Thus, experts may have overweighted the length of time in their assessments. It is therefore possible that some rules’ rankings based on the model were closer to the true condition than the rankings based on the experts.

The goal of this work was to produce a model that could be used for clinical decision support. However, to fully integrate such models into clinician workflow, clinicians must trust their output. “Black box” models, or those that are not readily interpretable by humans, present a challenge for trustworthiness [30]. It is often assumed that an increase in interpretability requires a tradeoff in terms of accuracy. However, rule ensembles (such as EAML) have been shown to be as or more accurate than high-performing models that are less interpretable, such as random forest and gradient boosting [22, 25]. EAML offers clinicians an opportunity to detect and correct problems before model deployment, which could increase their trust in model predictions and ultimately result in greater impact on patient care. For example, the misleading correlation between asthma and improved outcomes described above was discovered because one of the models built was rule-based [24]. A prior implementation of EAML detected a miscoded variable and a hidden confounder in the data [22]. In our study, no major issues were detected in the data upon reviewing the rules and delta between the expert and model rankings, reinforcing trustworthiness in our model.

A noteworthy advantage of EAML is its efficiency in expert quality assurance. Instead of manually reviewing thousands of observations with hundreds of features, experts assessed only the 46 rules selected by the model, each involving a small number of features. On average, reviewing each rule took less than 30 s. Rule-based ensembles could also improve interpretability in real-time by enabling clinicians to directly see in clinical decision support interfaces what rules apply to a given patient and how each influences a prediction shown.

A key limitation of this study is the lack of a secondary external data source. We were able to build a model for local use that penalized rules that had rankings discrepant with expert consensus. To optimally tune a model that was generalizable beyond the internal training and test sets to external sites, another dataset would be required. Without one, we were restricted to using the training set to regularize the EAML model. However, RuleFit had already selected the optimal rules and corresponding coefficients for the training set. Having an additional validation set would have enabled choosing the optimal subset of rules (hard EAML) or the amount of penalization to apply (soft EAML) to regularize the model most effectively for external datasets, while preserving an independent external test set on which to report performance. Given this constraint, we could only report performance on multiple models, rather than being able to select the best one. While PICUs A and B care for different patient populations, they are affiliated with the same academic medical center. This may have constrained our ability to demonstrate EAML’s full potential to generalize to new populations, although EAML’s improvement over RuleFit in the external test set was encouraging.

Another limitation of our model is that due to our binary outcome definition (successful extubation vs. all other outcomes, including no attempt made to extubate, failure, etc.), we were primarily able to predict when clinicians chose to extubate a patient rather than whether an extubation attempt would have been successful if it had been made. Additionally, our model does not account for factors such as time of day or unit staffing, which likely influence extubation timing given its elective nature. Building a model predicting extubation failure, an extremely rare event in our time window data structure, was not feasible. The primary outcome of interest for clinicians is the earliest time a patient could be successfully extubated to minimize complications from prolonged mechanical ventilation. While not directly identifiable in our data, we could potentially leverage variation among providers to identify subsets of patients who could be successfully extubated earlier. An alternative modeling approach would be to restrict the data to attempted extubations and explicitly predict extubation failure, but this would significantly reduce the number of observations available for training and render the model unable to learn effectively. Finally, the intubation and extubation data in the EHR required extensive cleaning; problems with accurately identifying timestamps for intubation and extubation have been previously reported and are not unique to our EHR [31]. Given that the comparisons between RuleFit and EAML were within datasets, however, this should not have affected our main conclusions.

Future studies that prospectively evaluate the model are essential next steps, which include running the model in the background of the EHR, and, if successful, and assessing its utility as clinical decision support for extubation readiness in real-time. The ultimate goal is to reduce ventilator-days and their associated harms, while ensuring extubation failures do not increase.

## Conclusions

EAML is a promising method for incorporating human expert knowledge into ML models, which may enhance clinician trust in ML model recommendations. This approach may be particularly useful in pediatric populations, which tend to be smaller in size, making it difficult to train site-specific models. EAML models show promise in improving the generalizability of models across sites without compromising robust performance. Future research should assess EAML’s impact on real-time decision-making and patient outcomes.

## Electronic supplementary material

Below is the link to the electronic supplementary material.

Supplementary Material 1

Supplementary Material 2

Supplementary Material 3

Supplementary Material 4

## Data Availability

The datasets analysed during the current study are not publicly available due to the presence of protected health information.

## Abbreviations

AUC: Area Under the Curve
BMI: Body Mass Index
CI: Confidence Interval
EAML: Expert-Augmented Machine Learning
EHR: Electronic Health Record
ETT: Endotracheal Tube
FiO2: Fraction of Inspired Oxygen
IQR: Inter-Quartile Range
LASSO: Least Absolute Shrinkage and Selection Operator
PEEP: Positive-End Expiratory Pressure
PICU: Pediatric Intensive Care Unit
ROC: Receiver Operating Characteristic
SBT: Spontaneous Breathing Trial
