# Supplementary material for: Expert-augmented machine learning for predicting extubation readiness in the pediatric intensive care unit
Source: BMC Med Inform Decis Mak. 2025 Jul 1;25:232. doi: 10.1186/s12911-025-03070-z (PMC12220236; doi:10.1186/s12911-025-03070-z)
Supplement: Supplementary file 1 — Supplementary Material 1 [file 12911_2025_3070_MOESM1_ESM.pdf]

Example patient timeline

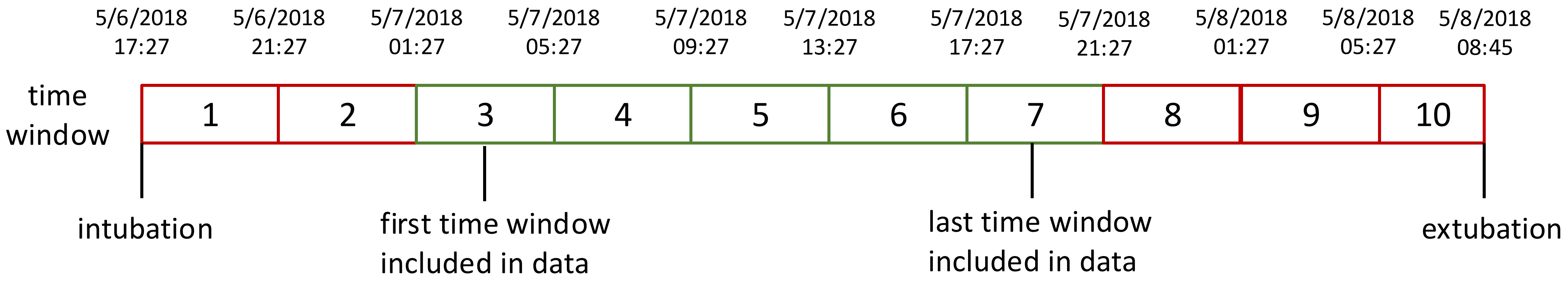

Patient data

Row	Start time	End time	Time window	Lagged variables: Time windows	Extubation status: Time window	Extubation status time
1	5/6/2018 01:27	5/6/2018 05:27	3	1 and 2	6	5/7/2018 17:27
2	5/6/2018 05:27	5/7/2018 09:27	4	2 and 3	7	5/7/2018 21:27
3	5/7/2018 09:27	5/7/2018 13:27	5	3 and 4	8	5/8/2018 01:27
4	5/7/2018 13:27	5/7/2018 17:27	6	4 and 5	9	5/8/2018 05:27
5	5/7/2018 17:27	5/7/2018 21:27	7	5 and 6	10	5/8/2018 08:45
