# Supplementary material for: Expert-augmented machine learning for predicting extubation readiness in the pediatric intensive care unit
Source: BMC Med Inform Decis Mak. 2025 Jul 1;25:232. doi: 10.1186/s12911-025-03070-z (PMC12220236; doi:10.1186/s12911-025-03070-z)
Supplement: Supplementary file 2 — Supplementary Material 2 [file 12911_2025_3070_MOESM2_ESM.pdf]

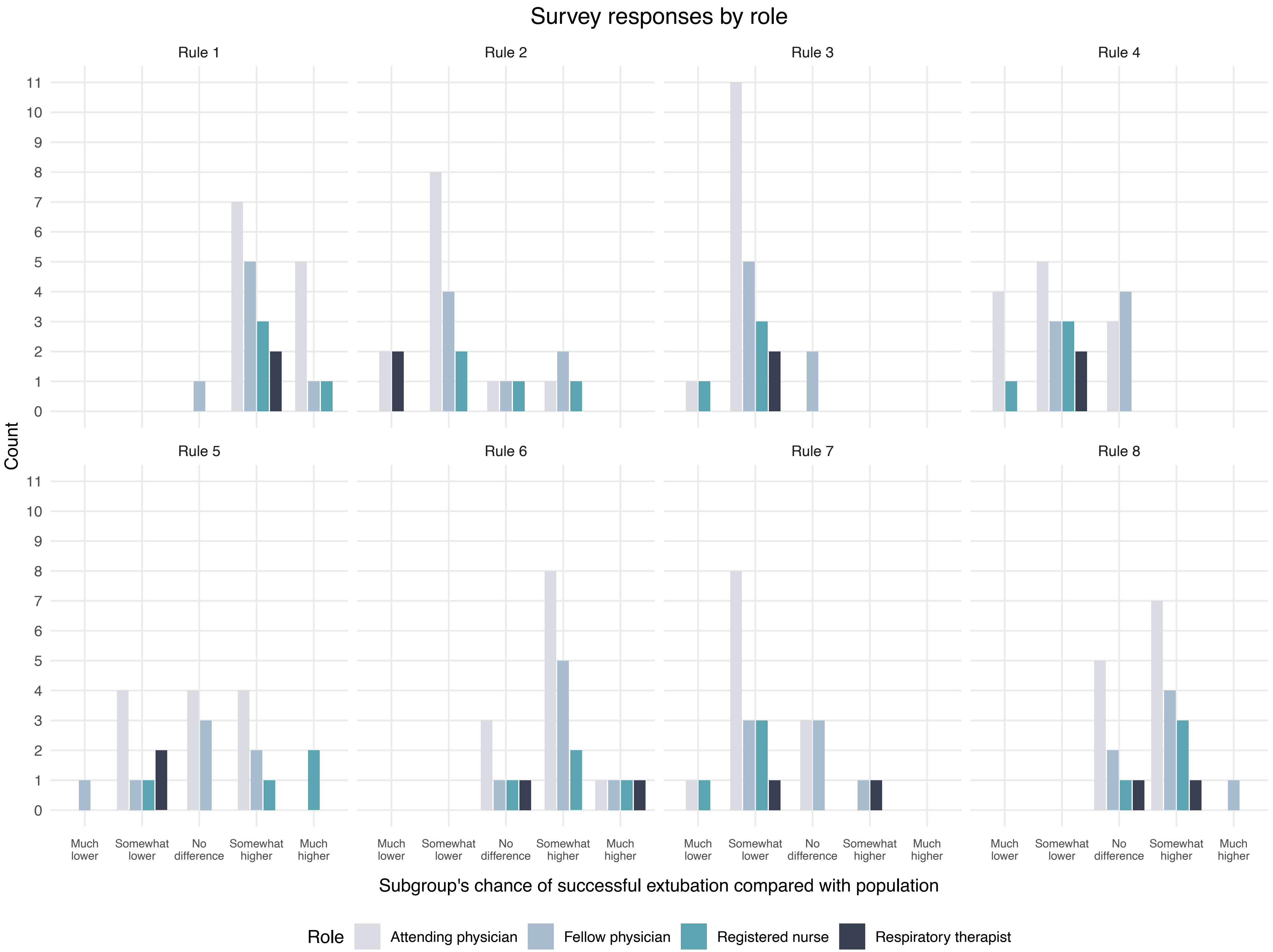

Survey responses by role
Rule 1
Rule 2
Rule 3
Rule 4
Rule 5
Rule 6
Rule 7
Rule 8
Count
Much lower
Somewhat lower
No difference
Somewhat higher
Much higher
Subgroup's chance of successful extubation compared with population
Role
Attending physician
Fellow physician
Registered nurse
Respiratory therapist

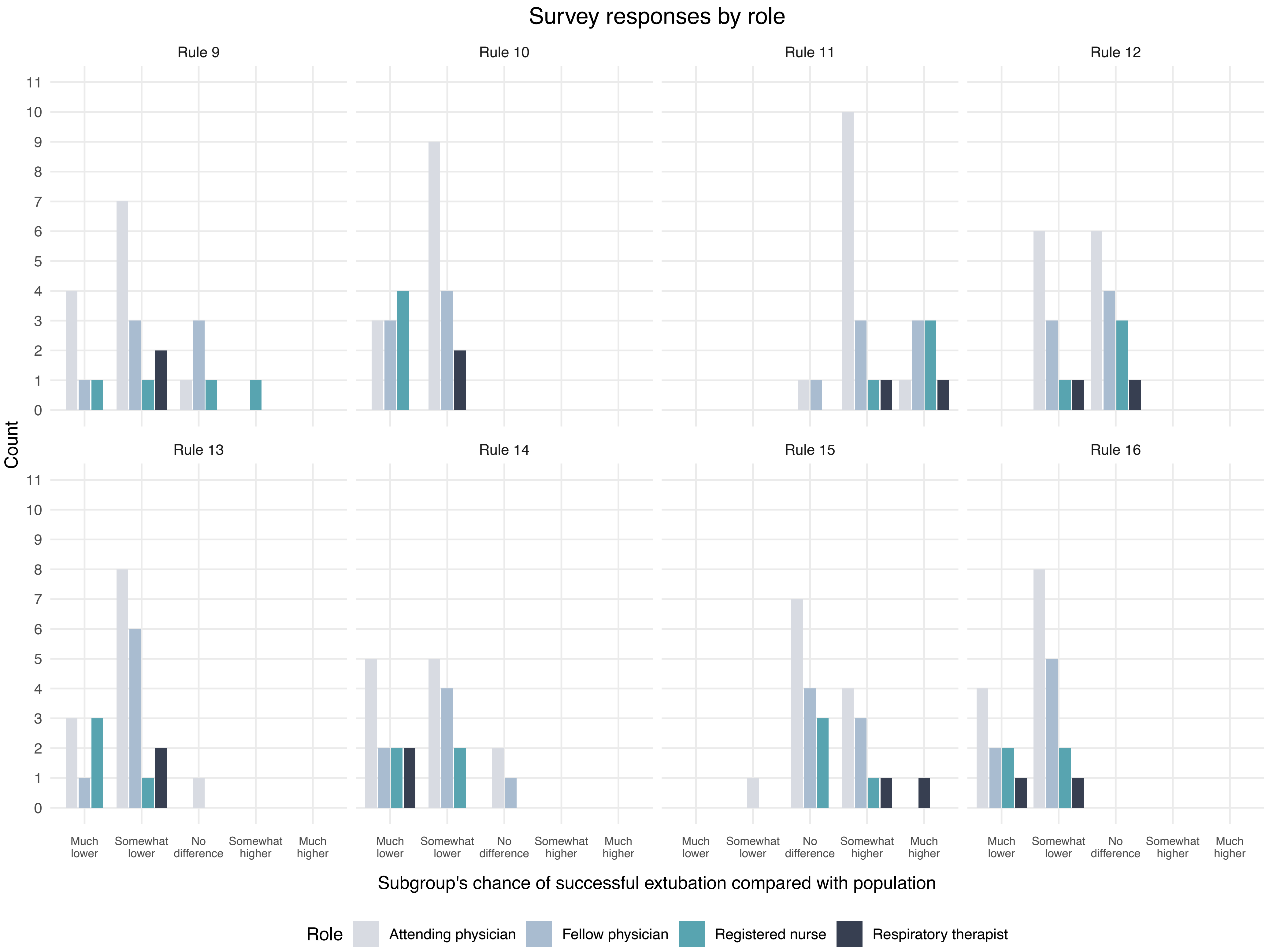
Survey responses by role
Rule 9
Rule 10
Rule 11
Rule 12
Rule 13
Rule 14
Rule 15
Rule 16
Count
0
1
2
3
4
5
6
7
8
9
10
11
Much lower
Somewhat lower
No difference
Somewhat higher
Much higher
Subgroup's chance of successful extubation compared with population
Role
Attending physician
Fellow physician
Registered nurse
Respiratory therapist

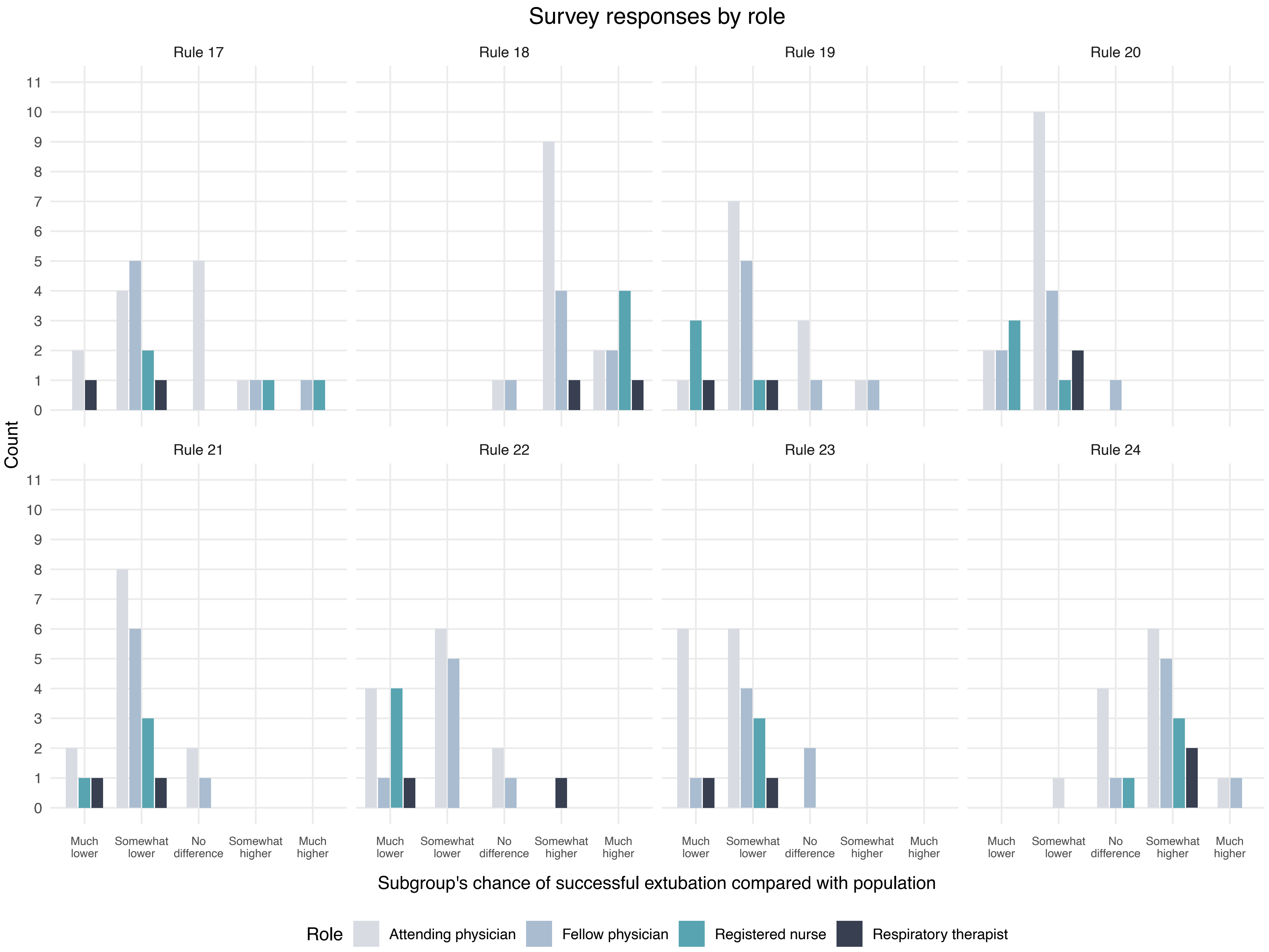

Survey responses by role
Rule 17
Rule 18
Rule 19
Rule 20
Rule 21
Rule 22
Rule 23
Rule 24
Count
0
1
2
3
4
5
6
7
8
9
10
11
Much lower
Somewhat lower
No difference
Somewhat higher
Much higher
Subgroup's chance of successful extubation compared with population
Role
Attending physician
Fellow physician
Registered nurse
Respiratory therapist

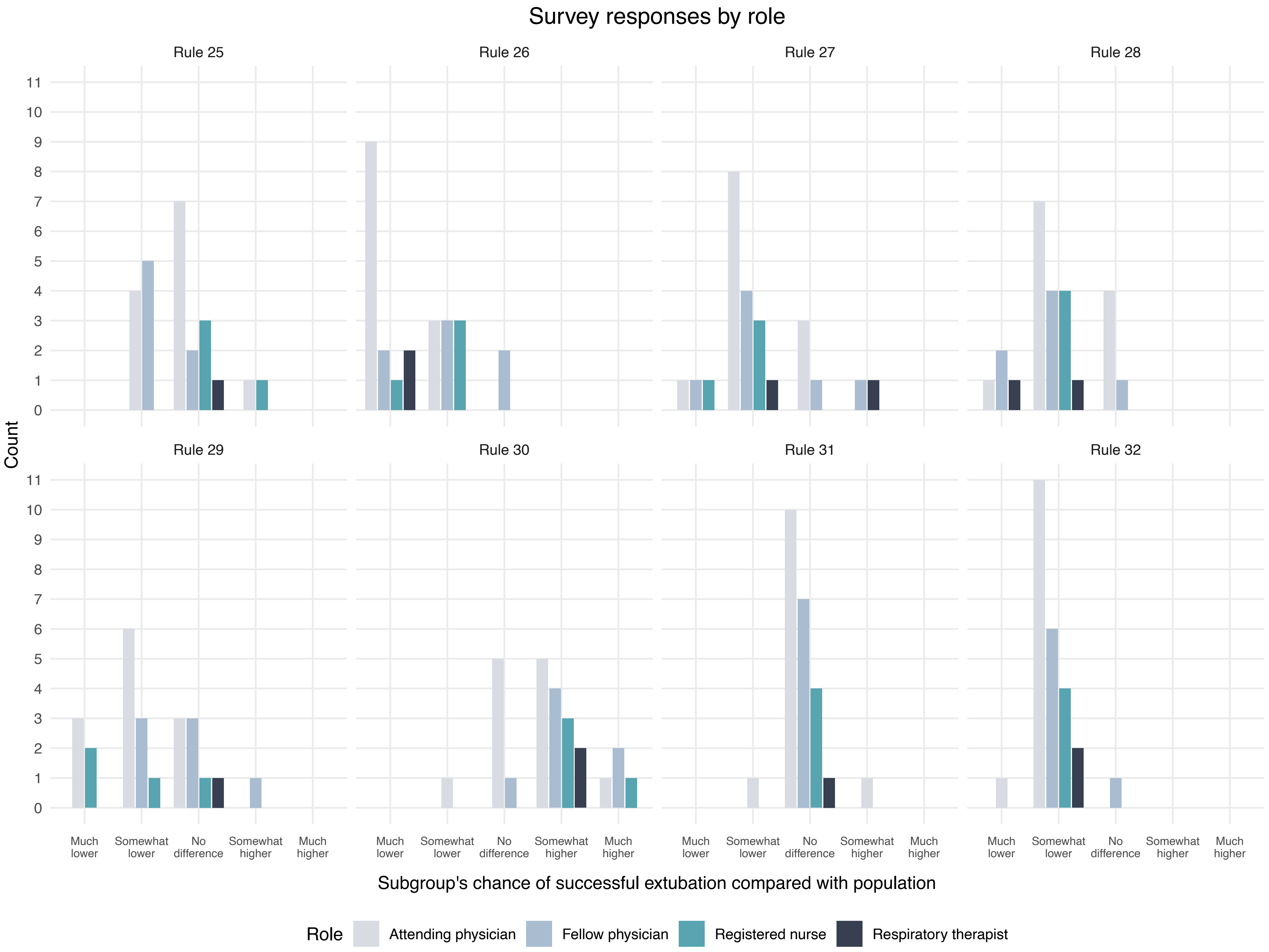
Survey responses by role
Rule 25
Rule 26
Rule 27
Rule 28
Rule 29
Rule 30
Rule 31
Rule 32
Count
Much lower
Somewhat lower
No difference
Somewhat higher
Much higher
Subgroup's chance of successful extubation compared with population
Role
Attending physician
Fellow physician
Registered nurse
Respiratory therapist

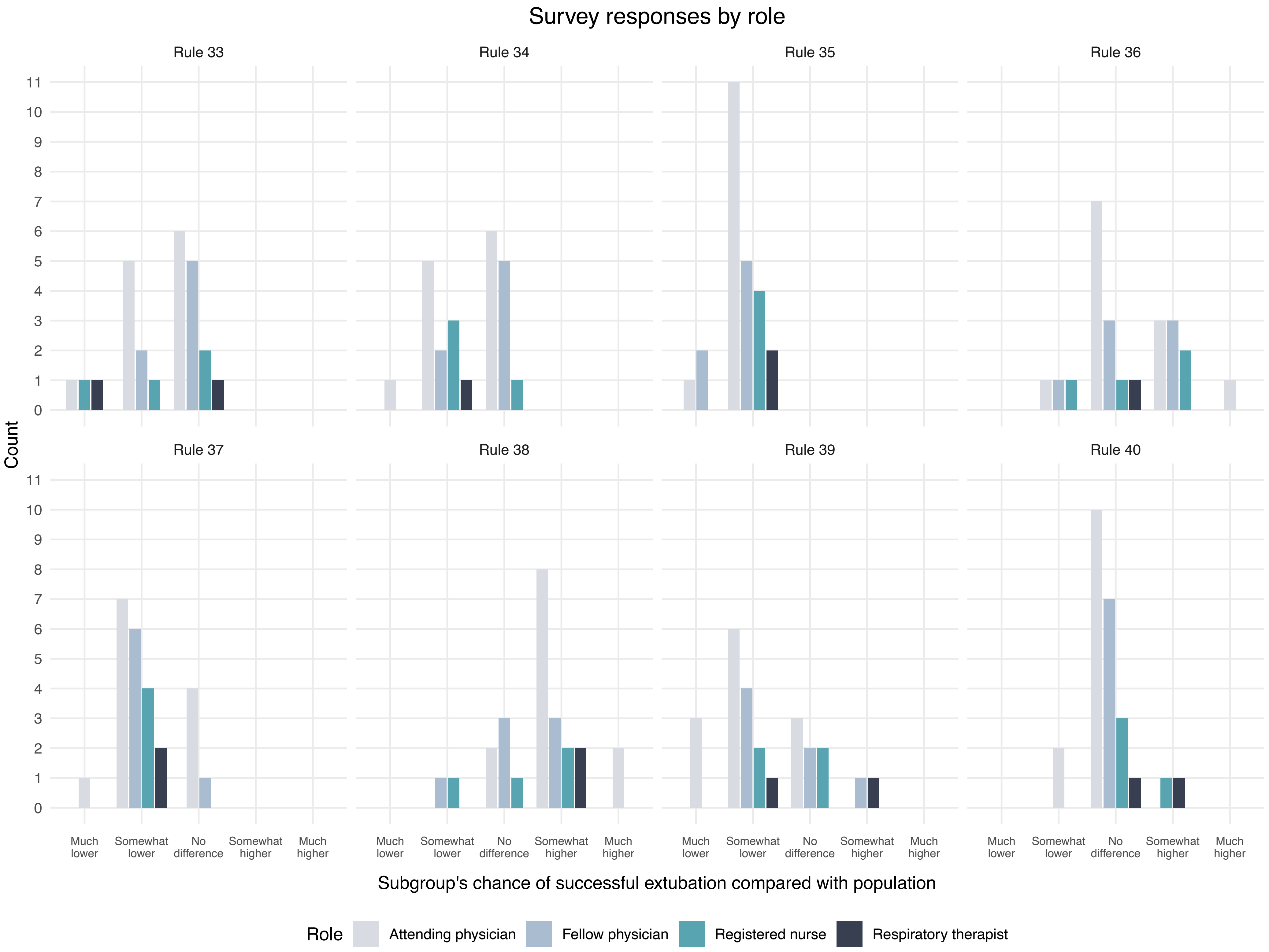

Survey responses by role
Rule 33
Rule 34
Rule 35
Rule 36
Rule 37
Rule 38
Rule 39
Rule 40
Count
Much lower
Somewhat lower
No difference
Somewhat higher
Much higher
Subgroup's chance of successful extubation compared with population
Role
Attending physician
Fellow physician
Registered nurse
Respiratory therapist

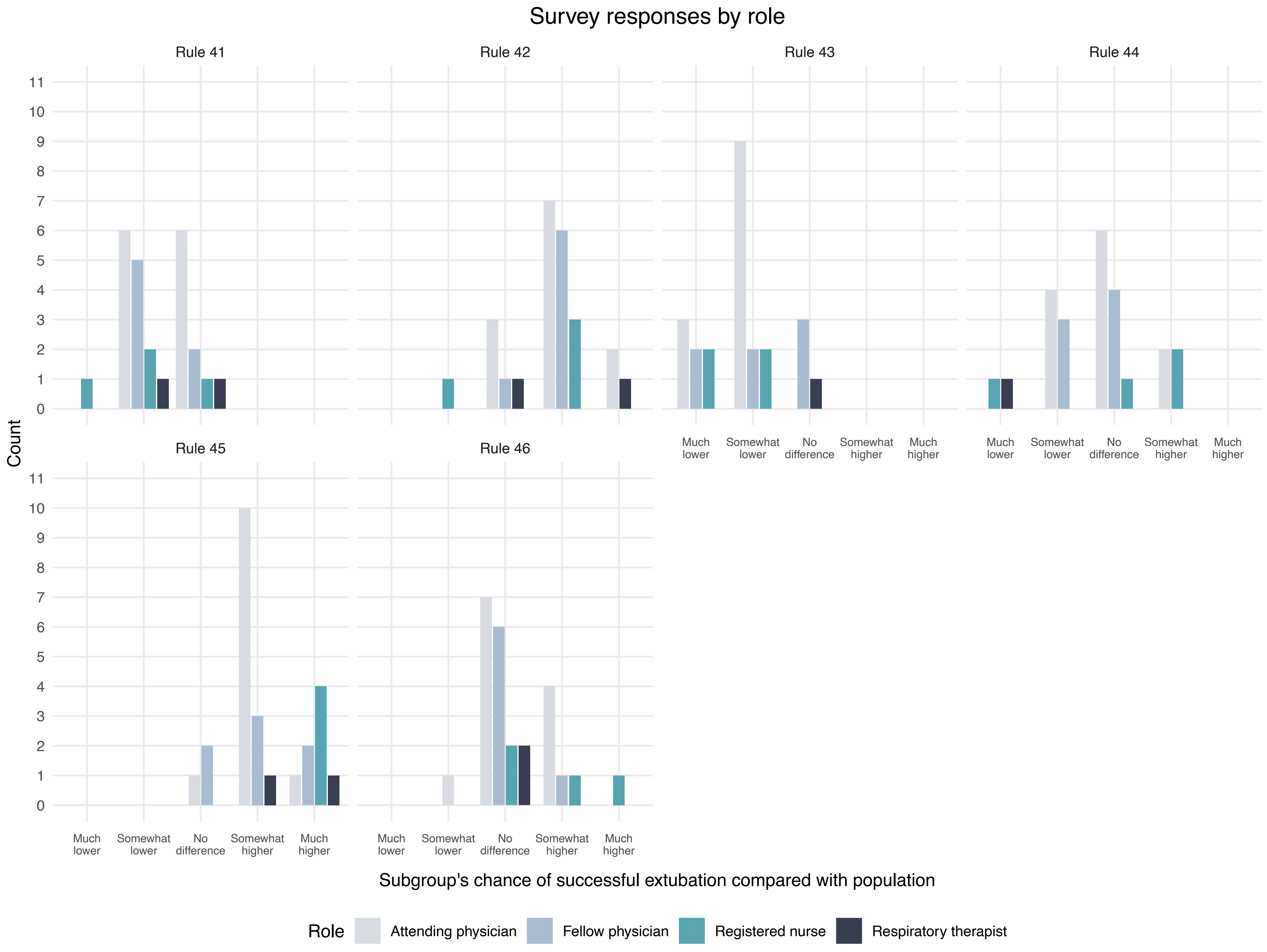

Survey responses by role
Rule 41
Rule 42
Rule 43
Rule 44
Rule 45
Rule 46
Count
0
1
2
3
4
5
6
7
8
9
10
11
Much lower
Somewhat lower
No difference
Somewhat higher
Much higher
Subgroup's chance of successful extubation compared with population
Role
Attending physician
Fellow physician
Registered nurse
Respiratory therapist

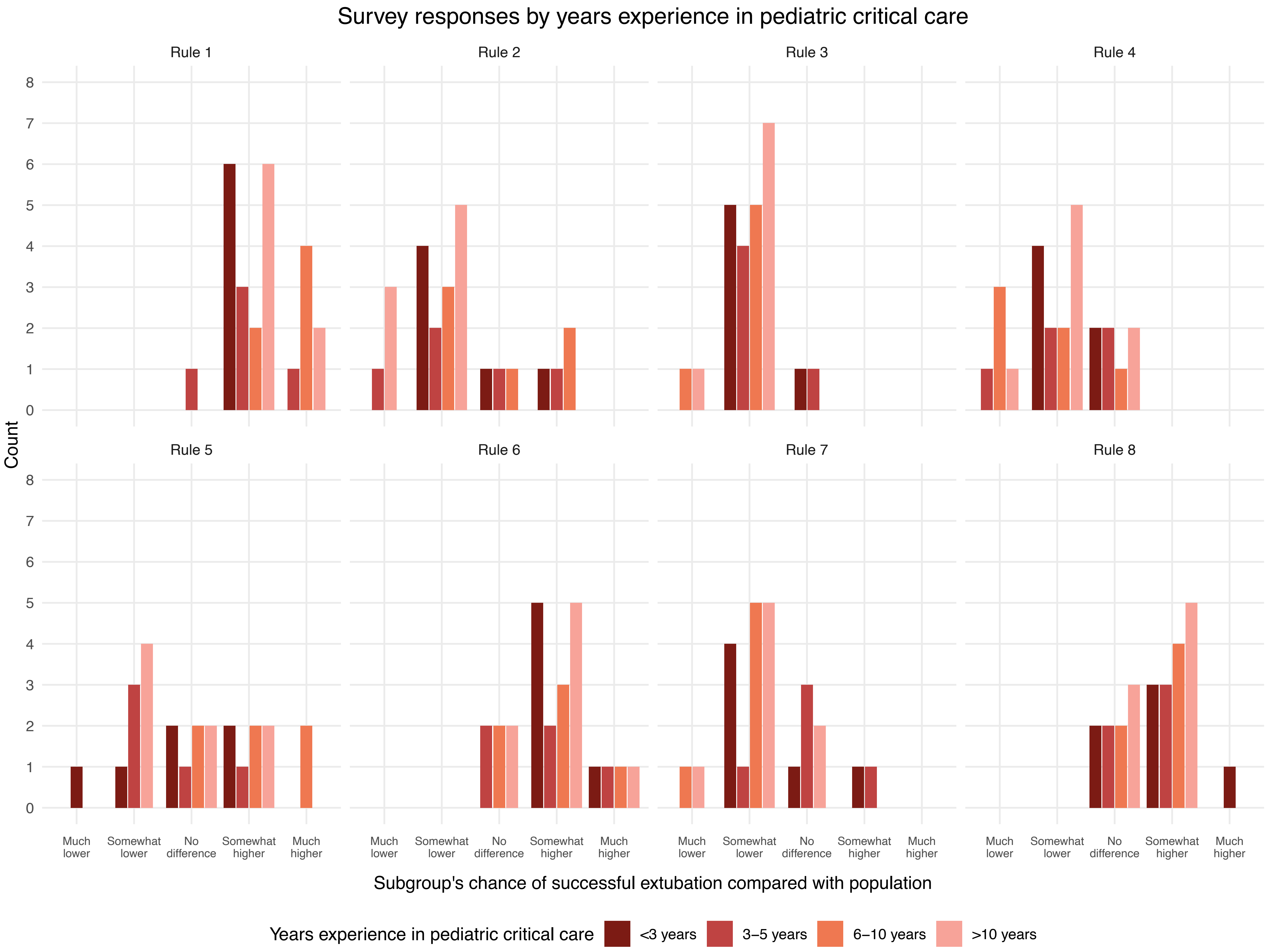

Survey responses by years experience in pediatric critical care
Rule 1
Rule 2
Rule 3
Rule 4
Rule 5
Rule 6
Rule 7
Rule 8
Count
Much lower
Somewhat lower
No difference
Somewhat higher
Much higher
Subgroup's chance of successful extubation compared with population
Years experience in pediatric critical care
<3 years
3–5 years
6–10 years
>10 years

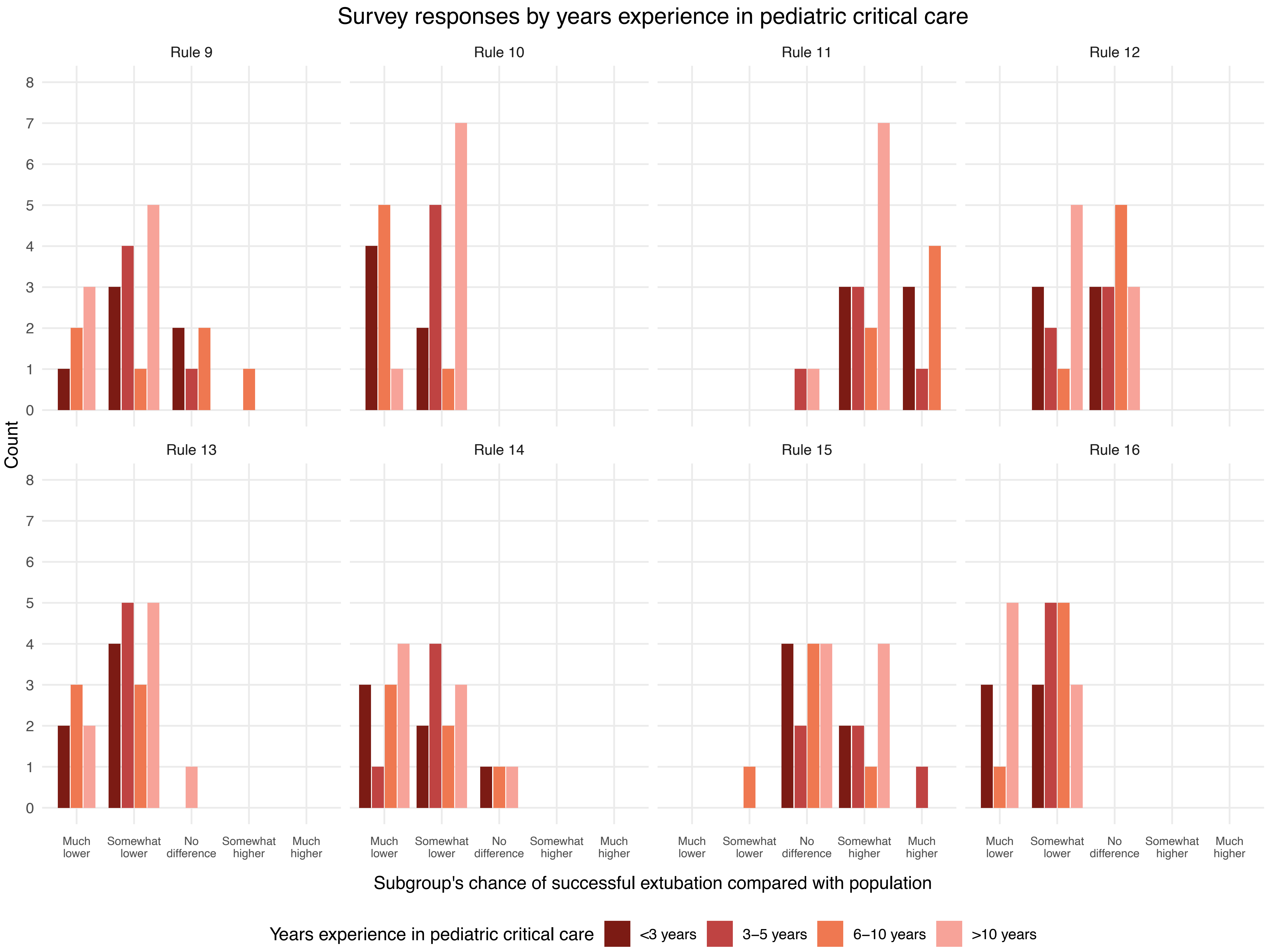

Survey responses by years experience in pediatric critical care
Rule 9
Rule 10
Rule 11
Rule 12
Rule 13
Rule 14
Rule 15
Rule 16
Count
Much lower
Somewhat lower
No difference
Somewhat higher
Much higher
Subgroup's chance of successful extubation compared with population
Years experience in pediatric critical care
<3 years
3–5 years
6–10 years
>10 years

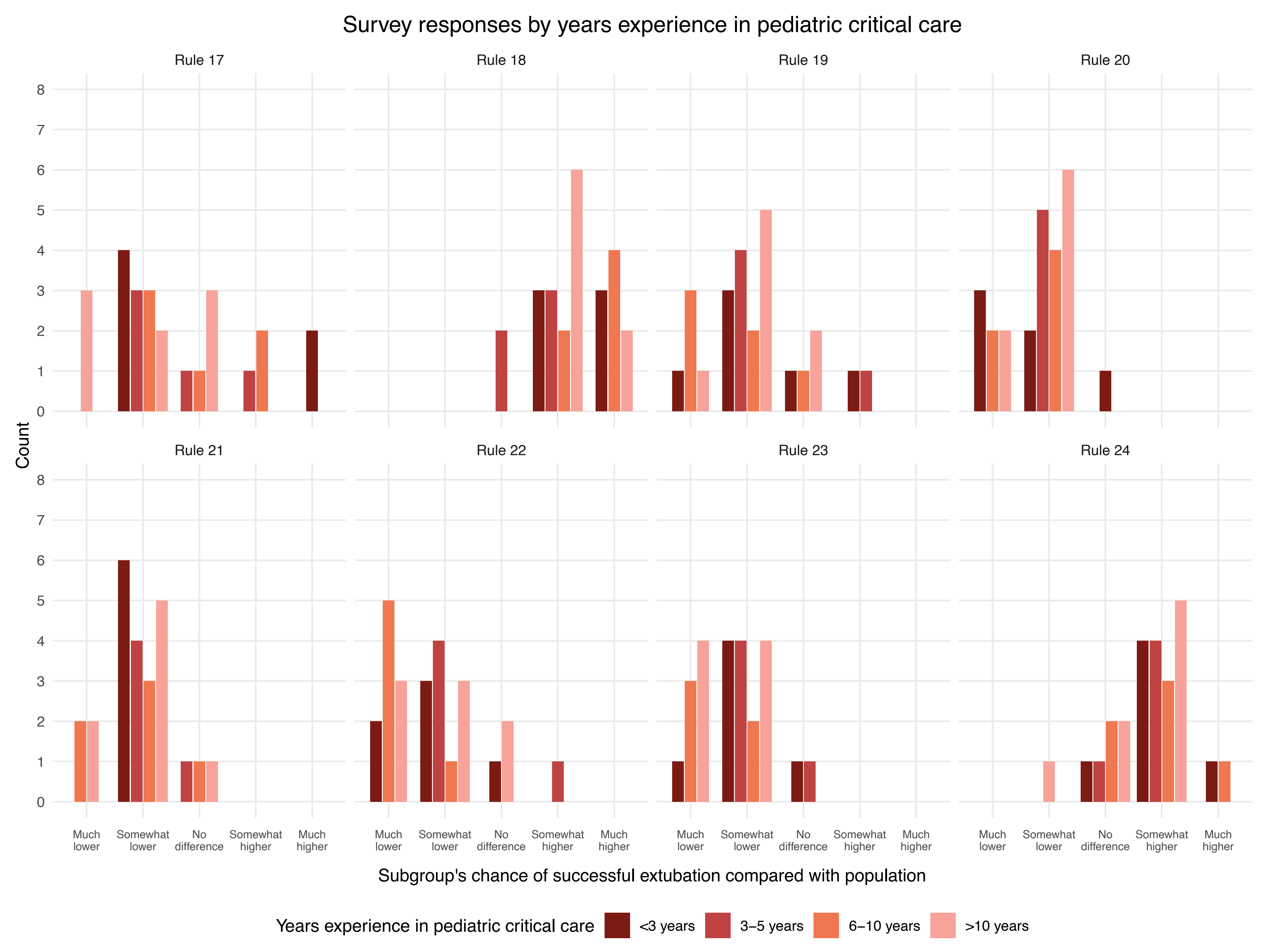

Survey responses by years experience in pediatric critical care
Rule 17
Rule 18
Rule 19
Rule 20
Rule 21
Rule 22
Rule 23
Rule 24
Count
0
1
2
3
4
5
6
7
8
Much lower
Somewhat lower
No difference
Somewhat higher
Much higher
Subgroup's chance of successful extubation compared with population
Years experience in pediatric critical care
<3 years
3–5 years
6–10 years
>10 years

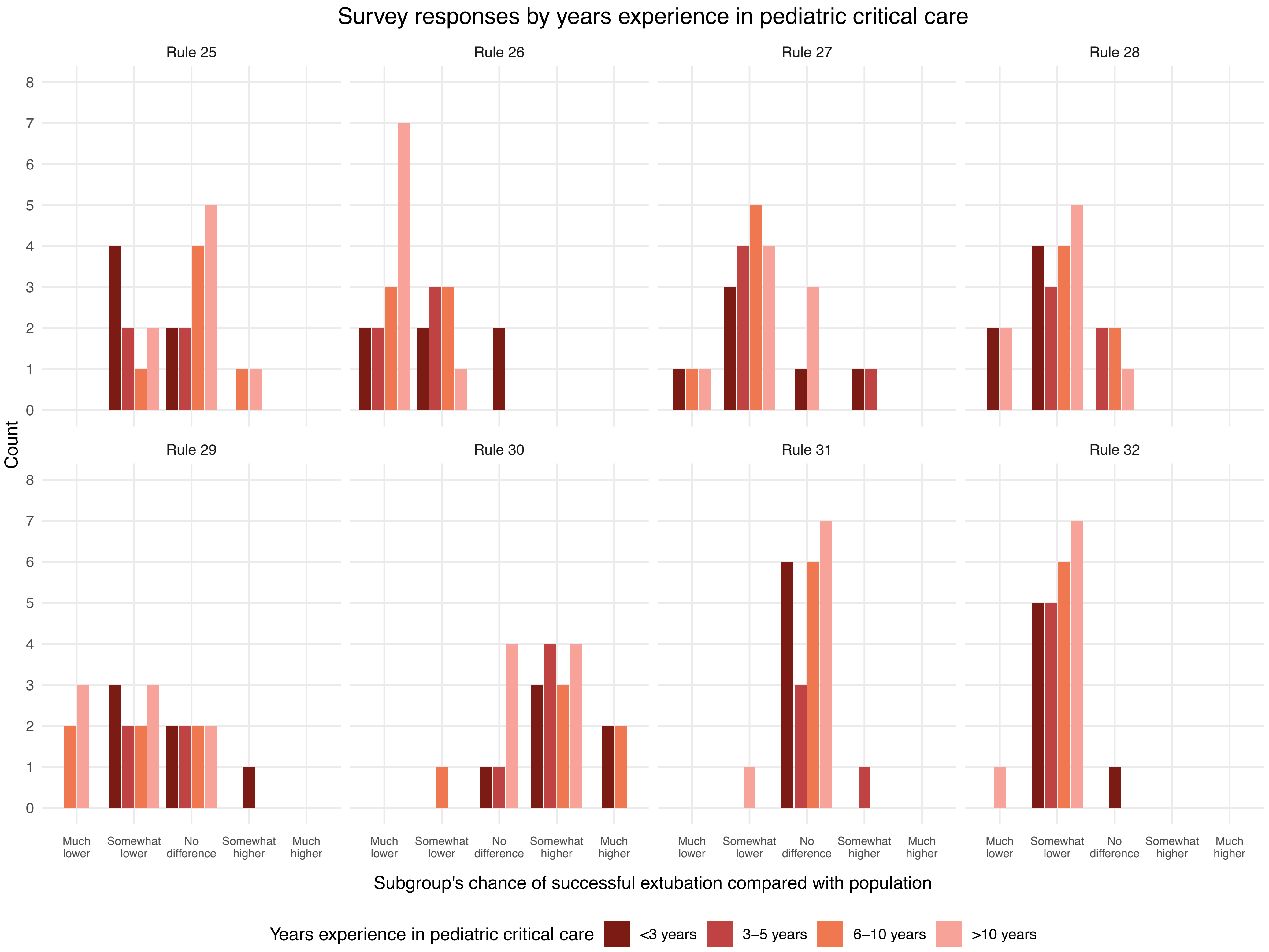
Survey responses by years experience in pediatric critical care
Rule 25
Rule 26
Rule 27
Rule 28
Rule 29
Rule 30
Rule 31
Rule 32
Count
Much lower
Somewhat lower
No difference
Somewhat higher
Much higher
Subgroup's chance of successful extubation compared with population
Years experience in pediatric critical care
<3 years
3–5 years
6–10 years
>10 years

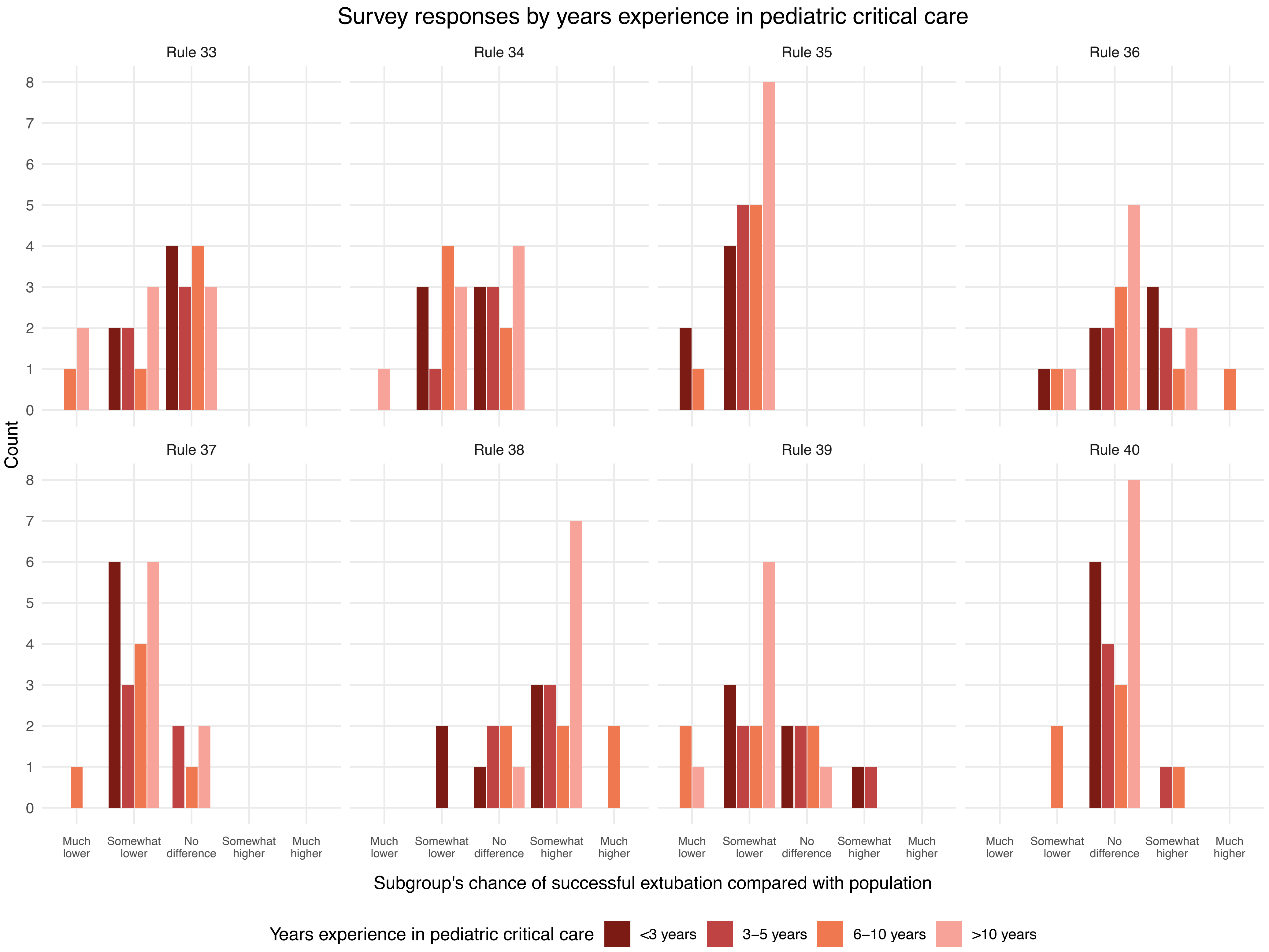

Survey responses by years experience in pediatric critical care
Rule 33
Rule 34
Rule 35
Rule 36
Rule 37
Rule 38
Rule 39
Rule 40
Count
Much lower
Somewhat lower
No difference
Somewhat higher
Much higher
Subgroup's chance of successful extubation compared with population
Years experience in pediatric critical care
<3 years
3–5 years
6–10 years
>10 years

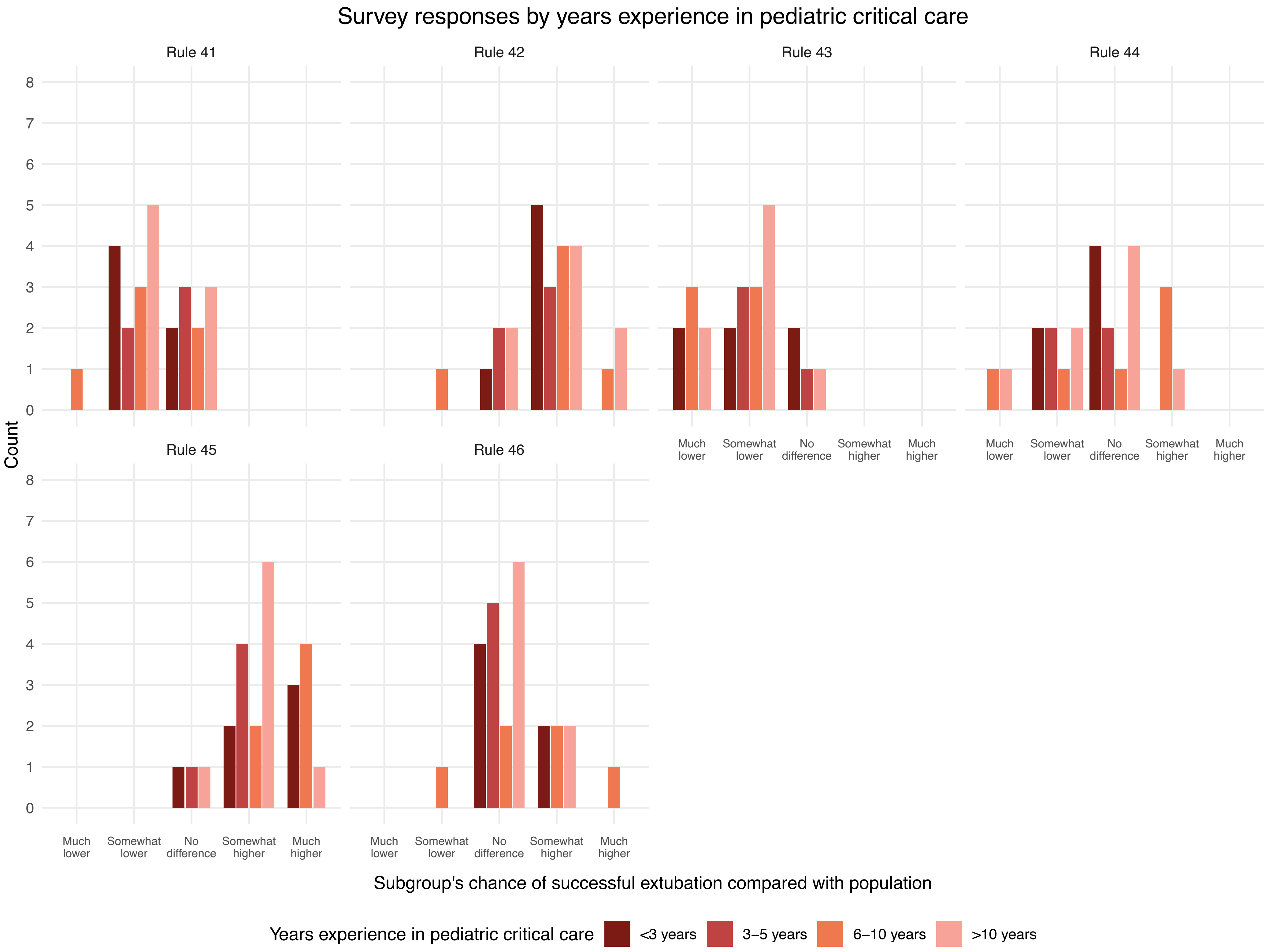

Survey responses by years experience in pediatric critical care
Rule 41
Rule 42
Rule 43
Rule 44
Rule 45
Rule 46
Count
Much lower
Somewhat lower
No difference
Somewhat higher
Much higher
Subgroup's chance of successful extubation compared with population
Years experience in pediatric critical care
<3 years
3–5 years
6–10 years
>10 years
