# Supplementary material for: Expert-augmented machine learning for predicting extubation readiness in the pediatric intensive care unit
Source: BMC Med Inform Decis Mak. 2025 Jul 1;25:232. doi: 10.1186/s12911-025-03070-z (PMC12220236; doi:10.1186/s12911-025-03070-z)
Supplement: Supplementary file 3 — Supplementary Material 3 [file 12911_2025_3070_MOESM3_ESM.pdf]

Supplementary Methods

Feature inclusion
We initially created a more extensive set of summary variables per time window: mean (numeric/binary), median (numeric/binary), mode (categorical), first value, last value, minimum value, and maximum value. To keep the most parsimonious set of variables for EAML without losing information, we estimated a cross-validated AUC in the training set of various combinations of summary variables using gradient boosted tree models (the first step of RuleFit). Mean/mode variables only performed as well as datasets with more summary variables.

Missing data
Missing values were not imputed as gradient boosted trees, the first step of RuleFit, do not require complete data. The LightGBM package, which is used in the LightRuleFit function with which we built our model, allocates missing values to the bins that optimally minimize loss.

Train/test split
We ensured that all time windows from each endotracheal tube (from intubation to extubation) were included only in the training data or the test data. When resampling the training data for tuning of model hyperparameters, we ensured that all observations from each endotracheal tube were kept within the same fold.

Outcome definition
The outcome for each time window reflects the patient's status 12 hours after the end of that window, calculated as the status after three subsequent 4-hour windows. However, because the start of each 4-hour window is defined by counting forward from the time of intubation, the final window may contain less than four hours if extubation does not coincide with multiples of four hours post-intubation (e.g., 24, 28, 32 hours). For instance, consider a patient intubated at 17:27 on 5/6/2018 and extubated at 08:45 on 5/8/2018 (Supplementary Figure 1). The last time window included in the model for this patient is from 17:27 to 21:27 on 5/7/2018. The subsequent windows, not included in the model, run from 5/7/2018 21:27 to 5/8/2018 01:27, from 01:27 to 05:27 on 5/8/2018, and from 05:27 to 08:45 on 5/8/2018. Since extubation occurred at the end of the third window, which was less than 12 hours after the end of the last included window, a more precise definition of the outcome is that it conveys the status 8.01 to 12 hours after the end of the last included time window.

Model tuning
For the LightGBM component of EAML, the learning rate (0.001, 0.01, 0.1) and number of leaves (4, 8, 16, 32) were tuned using exhaustive grid search. The number of trees is automatically tuned based on the validation error in the rtemis package. For the LASSO model, the glmnet package generates a sequence of lambdas; cv.glmnet performs cross-validation to find

the best lambda for each sub-sample and then the lambdas are averaged across all sub-samples as recommended by the glmnet authors.(1) Four stratified sub-samples (based on outcome) were used for all tuning; observations from each intubation were kept together in the resamples.

1. Friedman J, Hastie T, Tibshirani R. Regularization Paths for Generalized Linear Models via Coordinate Descent. J Stat Softw. 2010;33(1):1–22.

Survey: EAML Extubation Readiness

Start of Block: Introduction

Intro1 We are asking you to take part in a research study being done by Dr. Deborah Franzon and Jean Digitale at the University of California, San Francisco.

The purpose of this study is to gather human expert input for a machine learning model to **predict when patients in the pediatric intensive care unit will be ready for <u>successful extubation</u>**.

Being in this study is optional. If you choose to participate, you will complete a survey that asks you to rate whether certain clinical factors are associated with an increased chance of **<u>successful extubation</u>** 12 hours from now.

You can skip questions that you do not want to answer or stop the survey at any time. The survey is anonymous, and no one will be able to link your answers back to you. We expect it to take you <30 minutes.

Your input matters for patient care - we will use your responses to determine what model output makes sense from a clinical perspective. Our dataset consists of UCSF PICU patients from San Francisco and Oakland from 2013-2023. We plan to test this model or a similar one in our EHR to determine whether to implement as real-time clinical decision support.

Questions? Please contact Jean Digitale at jean.digitale@ucsf.edu. If you have questions or concerns about your rights as a research participant, you can call the UCSF Institutional Review Board at 415-476-1814. If you are willing to participate in this study, click the arrow button to start the survey.

Page Break

Intro2 **A few notes:**
The data is structured into 4-hour time windows (pulse, respiratory rate, and other clinical characteristics represent the average over the past 4 hours). To allow the model to incorporate changes over time, some variables represent averages from the prior time window (labeled: 4-8 hours ago) or two time windows ago (labeled: 8-12 hours ago).Each question includes a small subset of variables. The subgroups of patients overlap across questions. Treat each question individually.The clinical conditions may seem unrelated - they are directly output from the model.There are no correct or wrong answers.We know that you will not have all the information you would like to have about each subgroup, but given the information presented, give us your best assessment of how their **chances of successful extubation** 12 hours from now compare with all intubated PICU patients.

Now, let's look at some sample questions.

Page Break

Intro3 In each of the following questions, we will present a set of clinical conditions for a subgroup of PICU patients, as well as the entire PICU population intubated >24 hours. For numeric variables, median (minimum-maximum) is reported. For categorical variables, mode (all categories present) is reported.

An example is a subgroup that has a lower PEEP and a higher GCS on average than the intubated PICU population overall:

Ventilator:
PEEP (set)
Subgroup: 6 (5-7)
Population: 8 (5-19)

Other Clinical Data:
Glasgow coma scale score
Subgroup: 11 (7.5-15)
Population: 8 (3-15)

Then we will ask you:
Do patients in the subgroup have a higher or lower chance of successful extubation 12 hours from now compared with patients in the population?

Answer options:
Much higher
Somewhat higher
No difference
Somewhat lower
Much lower

Page Break

Intro4 Another example is a subgroup that was intubated on the day of a surgery compared with the full population, who most commonly were not intubated on the day of a surgery.

Remember: For categorical variables, mode is reported with all values present in the subgroup or population. Shown below, everyone in the subgroup was intubated on the day of a surgery; the only value present in the subgroup is "Yes". The most common value in the population is "No", but the population included individuals who were ("Yes") and were not ("No") intubated on the day of a surgery.

ETT:
ETT placed day of surgery
Subgroup: Yes (Yes)
Population: No (No, Yes)

Then we will ask you:
Do patients in the subgroup have a higher or lower chance of successful extubation 12 hours from now compared with patients in the population?

Answer options:
Much higher
Somewhat higher
No difference
Somewhat lower
Much lower

Page Break

End of Block: Introduction

Start of Block: Role

Role What is your professional role?

- Attending (1)
- Fellow (2)
- Resident (3)
- Nurse practitioner (4)
- Physician assistant (5)
- Registered nurse (6)
- Respiratory therapist (7)
- Other (8)

Page Break

Years How many years of experience do you have working in pediatric critical care?

- ◯ <3 years (1)
- ◯ 3-5 years (2)
- ◯ 6-10 years (3)
- ◯ >10 years (4)

End of Block: Role

Start of Block: Imported Block 1 - Feb 7, 2024

1
For numeric variables, median (minimum, maximum) is reported. For categorical variables, mode (all values present) is reported.

Ventilator:
Mean airway pressure (measured)
Subgroup: 8 (3, 11.1)
Population: 11 (3, 35.5)

PEEP (set)
Subgroup: 5 (5, 5)
Population: 7 (5, 19)

Other Clinical Data:
Glasgow coma scale score: 4-8 hours ago
Subgroup: 9 (5.3, 15)
Population: 8 (3, 15)

Do patients in the subgroup have a higher or lower chance of **successful extubation** 12 hours

from now compared with patients in the population?

- ○ Much higher (1)
- ○ Somewhat higher (2)
- ○ No difference (3)
- ○ Somewhat lower (4)
- ○ Much lower (5)

Page Break

2
For numeric variables, median (minimum, maximum) is reported. For categorical variables, mode (all values present) is reported.

Ventilator:
PEEP (set)
Subgroup: 5 (5, 6.5)
Population: 7 (5, 19)

Mean airway pressure (measured): 4-8 hours ago
Subgroup: 8 (3, 11)
Population: 11 (3, 35.5)

Other Clinical Data:
Glasgow coma scale score
Subgroup: 5 (3, 7.8)
Population: 8 (3, 15)

Do patients in the subgroup have a higher or lower chance of **successful extubation** 12 hours from now compared with patients in the population?

○ Much higher (1)

○ Somewhat higher (2)

○ No difference (3)

○ Somewhat lower (4)

○ Much lower (5)

Page Break

3
For numeric variables, median (minimum, maximum) is reported. For categorical variables, mode (all values present) is reported.

Ventilator:
PEEP (set)
Subgroup: 9 (6.7, 19)
Population: 7 (5, 19)

Do patients in the subgroup have a higher or lower chance of **successful extubation** 12 hours from now compared with patients in the population?

○ Much higher (1)

○ Somewhat higher (2)

○ No difference (3)

○ Somewhat lower (4)

○ Much lower (5)

Page Break

4
For numeric variables, median (minimum, maximum) is reported. For categorical variables, mode (all values present) is reported.

Ventilator:
Mean airway pressure (measured): 4-8 hours ago
Subgroup: 14 (11.2, 35.5)
Population: 11 (3, 35.5)

Do patients in the subgroup have a higher or lower chance of **successful extubation** 12 hours from now compared with patients in the population?

○ Much higher (1)

○ Somewhat higher (2)

○ No difference (3)

○ Somewhat lower (4)

○ Much lower (5)

Page Break

5
For numeric variables, median (minimum, maximum) is reported. For categorical variables, mode (all values present) is reported.

Ventilator:
PEEP (set)
Subgroup: 5 (5, 7.2)
Population: 7 (5, 19)

Mean airway pressure (measured)
Subgroup: 8.1 (3, 10)
Population: 11 (3, 35.5)

Other Clinical Data:
Average lower extremity motor response (0 = no movement, 5 = full power)
Subgroup: 0 (0, 0.6)
Population: 2 (0, 5)

Do patients in the subgroup have a higher or lower chance of **successful extubation** 12 hours from now compared with patients in the population?

○ Much higher (1)

○ Somewhat higher (2)

○ No difference (3)

○ Somewhat lower (4)

○ Much lower (5)

Page Break

6
For numeric variables, median (minimum, maximum) is reported. For categorical variables, mode (all values present) is reported.

Ventilator:
Mean airway pressure (measured)
Subgroup: 8.5 (3, 10.1)
Population: 11 (3, 35.5)

Do patients in the subgroup have a higher or lower chance of **successful extubation** 12 hours from now compared with patients in the population?

- ○ Much higher (1)
- ○ Somewhat higher (2)
- ○ No difference (3)
- ○ Somewhat lower (4)
- ○ Much lower (5)

Page Break

7
For numeric variables, median (minimum, maximum) is reported. For categorical variables, mode (all values present) is reported.

Ventilator:
Mean airway pressure (measured)
Subgroup: 14 (11.5, 35.5)
Population: 11 (3, 35.5)

Do patients in the subgroup have a higher or lower chance of **successful extubation** 12 hours from now compared with patients in the population?

○ Much higher (1)

○ Somewhat higher (2)

○ No difference (3)

○ Somewhat lower (4)

○ Much lower (5)

Page Break

8

For numeric variables, median (minimum, maximum) is reported. For categorical variables, mode (all values present) is reported.

Vital Signs:

Pulse percentile (age-adjusted)

Subgroup: 45.1 (0, 76.5)

Population: 69.6 (0, 100)

Ventilator:

PEEP (set)

Subgroup: 5 (5, 7.2)

Population: 7 (5, 19)

Respiratory rate (set)

Subgroup: 18 (0, 27.5)

Population: 20 (0, 60)

Do patients in the subgroup have a higher or lower chance of **successful extubation** 12 hours from now compared with patients in the population?

- ○ Much higher (1)
- ○ Somewhat higher (2)
- ○ No difference (3)
- ○ Somewhat lower (4)
- ○ Much lower (5)

Page Break

9
For numeric variables, median (minimum, maximum) is reported. For categorical variables, mode (all values present) is reported.

Ventilator:
Mean airway pressure (measured): 4-8 hours ago
Subgroup: 9 (3, 11.2)
Population: 11 (3, 35.5)

Other Clinical Data:
Average upper extremity motor response (0 = no movement, 5 = full power)
Subgroup: 0 (0, 1.2)
Population: 2.5 (0, 5)

Do patients in the subgroup have a higher or lower chance of **successful extubation** 12 hours from now compared with patients in the population?

○ Much higher (1)

○ Somewhat higher (2)

○ No difference (3)

○ Somewhat lower (4)

○ Much lower (5)

Page Break

10

For numeric variables, median (minimum, maximum) is reported. For categorical variables, mode (all values present) is reported.

Ventilator:

PEEP (set)

Subgroup: 10 (6.7, 19)

Population: 7 (5, 19)

Mean airway pressure (measured)

Subgroup: 15 (11.4, 35)

Population: 11 (3, 35.5)

Do patients in the subgroup have a higher or lower chance of **successful extubation** 12 hours from now compared with patients in the population?

- ○ Much higher (1)
- ○ Somewhat higher (2)
- ○ No difference (3)
- ○ Somewhat lower (4)
- ○ Much lower (5)

Page Break

11

For numeric variables, median (minimum, maximum) is reported. For categorical variables, mode (all values present) is reported.

Ventilator:

PEEP (set): 4-8 hours ago

Subgroup: 5 (5, 8.5)

Population: 7 (5, 19)

Peak inspiratory pressure (measured)

Subgroup: 17 (6.6, 21.2)

Population: 21.2 (5.7, 56)

Other Clinical Data:

Average lower extremity motor response (0 = no movement, 5 = full power): 4-8 hours ago

Subgroup: 3 (0.8, 5)

Population: 2 (0, 5)

Do patients in the subgroup have a higher or lower chance of **successful extubation** 12 hours from now compared with patients in the population?

- ◯ Much higher (1)
- ◯ Somewhat higher (2)
- ◯ No difference (3)
- ◯ Somewhat lower (4)
- ◯ Much lower (5)

Page Break

12

For numeric variables, median (minimum, maximum) is reported. For categorical variables, mode (all values present) is reported.

Ventilator:

PEEP (set): 4-8 hours ago

Subgroup: 7 (5, 8.5)

Population: 7 (5, 19)

Peak inspiratory pressure (measured)

Subgroup: 25 (21.2, 56)

Population: 21.2 (5.7, 56)

Other Clinical Data:

Average lower extremity motor response (0 = no movement, 5 = full power): 4-8 hours ago

Subgroup: 3 (0.8, 5)

Population: 2 (0, 5)

Do patients in the subgroup have a higher or lower chance of **successful extubation** 12 hours from now compared with patients in the population?

- ○ Much higher (1)
- ○ Somewhat higher (2)
- ○ No difference (3)
- ○ Somewhat lower (4)
- ○ Much lower (5)

Page Break

13

For numeric variables, median (minimum, maximum) is reported. For categorical variables, mode (all values present) is reported.

Ventilator:

PEEP (set): 4-8 hours ago

Subgroup: 10 (8.7, 19)

Population: 7 (5, 19)

Do patients in the subgroup have a higher or lower chance of **successful extubation** 12 hours from now compared with patients in the population?

○ Much higher (1)

○ Somewhat higher (2)

○ No difference (3)

○ Somewhat lower (4)

○ Much lower (5)

Page Break

14

For numeric variables, median (minimum, maximum) is reported. For categorical variables, mode (all values present) is reported.

Ventilator:

Mean airway pressure (measured): 4-8 hours ago

Subgroup: 9 (3, 11.7)

Population: 11 (3, 35.5)

Other Clinical Data:

Glasgow coma scale score: 8-12 hours ago

Subgroup: 4 (3, 6.7)

Population: 8 (3, 15)

Do patients in the subgroup have a higher or lower chance of **successful extubation** 12 hours from now compared with patients in the population?

- ○ Much higher (1)
- ○ Somewhat higher (2)
- ○ No difference (3)
- ○ Somewhat lower (4)
- ○ Much lower (5)

Page Break

15

For numeric variables, median (minimum, maximum) is reported. For categorical variables, mode (all values present) is reported.

Ventilator:

Mean airway pressure (measured): 4-8 hours ago

Subgroup: 12.6 (11.8, 34)

Population: 11 (3, 35.5)

Peak inspiratory pressure (measured)

Subgroup: 16 (7, 17)

Population: 21.2 (5.7, 56)

Do patients in the subgroup have a higher or lower chance of **successful extubation** 12 hours from now compared with patients in the population?

○ Much higher (1)

○ Somewhat higher (2)

○ No difference (3)

○ Somewhat lower (4)

○ Much lower (5)

Page Break

16

For numeric variables, median (minimum, maximum) is reported. For categorical variables, mode (all values present) is reported.

Vital Signs:

End-tidal CO2: 8-12 hours ago

Subgroup: 53.2 (47.2, 100)

Population: 41.9 (1, 100)

Ventilator:

PEEP (set)

Subgroup: 10 (6.7, 18)

Population: 7 (5, 19)

Do patients in the subgroup have a higher or lower chance of **successful extubation** 12 hours from now compared with patients in the population?

- ○ Much higher (1)
- ○ Somewhat higher (2)
- ○ No difference (3)
- ○ Somewhat lower (4)
- ○ Much lower (5)

Page Break

17
For numeric variables, median (minimum, maximum) is reported. For categorical variables, mode (all values present) is reported.

ETT:
Hours intubated
Subgroup: 64 (12, 208)
Population: 132 (12, 3072)

Ventilator:
PEEP (set): 4-8 hours ago
Subgroup: 6 (5, 9)
Population: 7 (5, 19)

Other Clinical Data:
Glasgow coma scale score
Subgroup: 5 (3, 7.8)
Population: 8 (3, 15)

Do patients in the subgroup have a higher or lower chance of **successful extubation** 12 hours from now compared with patients in the population?

- ○ Much higher (1)
- ○ Somewhat higher (2)
- ○ No difference (3)
- ○ Somewhat lower (4)
- ○ Much lower (5)

Page Break

18
For numeric variables, median (minimum, maximum) is reported. For categorical variables, mode (all values present) is reported.

ETT:
Hours intubated
Subgroup: 76 (12, 208)
Population: 132 (12, 3072)

Ventilator:
PEEP (set): 4-8 hours ago
Subgroup: 6 (5, 9)
Population: 7 (5, 19)

Other Clinical Data:
Glasgow coma scale score
Subgroup: 10.2 (8, 15)
Population: 8 (3, 15)

Do patients in the subgroup have a higher or lower chance of **successful extubation** 12 hours from now compared with patients in the population?

○ Much higher (1)

○ Somewhat higher (2)

○ No difference (3)

○ Somewhat lower (4)

○ Much lower (5)

Page Break

19

For numeric variables, median (minimum, maximum) is reported. For categorical variables, mode (all values present) is reported.

ETT:

Hours intubated

Subgroup: 412 (212, 3068)

Population: 132 (12, 3072)

Ventilator:

PEEP (set): 4-8 hours ago

Subgroup: 6 (5, 9)

Population: 7 (5, 19)

Do patients in the subgroup have a higher or lower chance of **successful extubation** 12 hours from now compared with patients in the population?

○ Much higher (1)

○ Somewhat higher (2)

○ No difference (3)

○ Somewhat lower (4)

○ Much lower (5)

Page Break

20
For numeric variables, median (minimum, maximum) is reported. For categorical variables, mode (all values present) is reported.

Ventilator:
PEEP (set): 4-8 hours ago
Subgroup: 10 (9.2, 19)
Population: 7 (5, 19)

Do patients in the subgroup have a higher or lower chance of **successful extubation** 12 hours from now compared with patients in the population?

- ○ Much higher (1)
- ○ Somewhat higher (2)
- ○ No difference (3)
- ○ Somewhat lower (4)
- ○ Much lower (5)

Page Break

21
For numeric variables, median (minimum, maximum) is reported. For categorical variables, mode (all values present) is reported.

ETT:
Hours intubated
Subgroup: 120 (12, 332)
Population: 132 (12, 3072)

Ventilator:
PEEP (set): 4-8 hours ago
Subgroup: 7 (5, 9)
Population: 7 (5, 19)

Labs:
pCO2
Subgroup: 63 (57.7, 109.3)
Population: 46 (17, 110.3)

Do patients in the subgroup have a higher or lower chance of **successful extubation** 12 hours from now compared with patients in the population?

○ Much higher (1)

○ Somewhat higher (2)

○ No difference (3)

○ Somewhat lower (4)

○ Much lower (5)

Page Break

22
For numeric variables, median (minimum, maximum) is reported. For categorical variables, mode (all values present) is reported.

ETT:
Hours intubated
Subgroup: 612 (336, 3068)
Population: 132 (12, 3072)

Ventilator:
PEEP (set): 4-8 hours ago
Subgroup: 6 (5, 9)
Population: 7 (5, 19)

Do patients in the subgroup have a higher or lower chance of **successful extubation** 12 hours from now compared with patients in the population?

- ○ Much higher (1)
- ○ Somewhat higher (2)
- ○ No difference (3)
- ○ Somewhat lower (4)
- ○ Much lower (5)

Page Break

23
For numeric variables, median (minimum, maximum) is reported. For categorical variables, mode (all values present) is reported.

Vital Signs:
Pulse percentile (age-adjusted)
Subgroup: 39.7 (0.1, 69.5)
Population: 69.6 (0, 100)

Other Clinical Data:
Glasgow coma scale score: 4-8 hours ago
Subgroup: 3.8 (3, 7)
Population: 8 (3, 15)

BMI z-score/Weight-for-length z-score: Baseline
Subgroup: -1.2 (-11.2, -0.1)
Population: 0.5 (-11.2, 7.6)

Do patients in the subgroup have a higher or lower chance of **successful extubation** 12 hours from now compared with patients in the population?

○ Much higher (1)

○ Somewhat higher (2)

○ No difference (3)

○ Somewhat lower (4)

○ Much lower (5)

Page Break

24
For numeric variables, median (minimum, maximum) is reported. For categorical variables, mode (all values present) is reported.

Vital Signs:
Pulse percentile (age-adjusted)
Subgroup: 39.5 (0, 69.5)
Population: 69.6 (0, 100)

Other Clinical Data:
Glasgow coma scale score: 4-8 hours ago
Subgroup: 11 (7.2, 15)
Population: 8 (3, 15)

Do patients in the subgroup have a higher or lower chance of **successful extubation** 12 hours from now compared with patients in the population?

○ Much higher (1)

○ Somewhat higher (2)

○ No difference (3)

○ Somewhat lower (4)

○ Much lower (5)

Page Break

25

For numeric variables, median (minimum, maximum) is reported. For categorical variables, mode (all values present) is reported.

Vital Signs:

Pulse percentile (age-adjusted)

Subgroup: 87.9 (69.5, 100)

Population: 69.6 (0, 100)

Do patients in the subgroup have a higher or lower chance of **successful extubation** 12 hours from now compared with patients in the population?

- ○ Much higher (1)
- ○ Somewhat higher (2)
- ○ No difference (3)
- ○ Somewhat lower (4)
- ○ Much lower (5)

Page Break

26
For numeric variables, median (minimum, maximum) is reported. For categorical variables, mode (all values present) is reported.

Ventilator:
Respiratory rate (set)
Subgroup: 18 (2, 27.5)
Population: 20 (0, 60)

Other Clinical Data:
Glasgow coma scale score: 8-12 hours ago
Subgroup: 3 (3, 4.8)
Population: 8 (3, 15)

Do patients in the subgroup have a higher or lower chance of **successful extubation** 12 hours from now compared with patients in the population?

- ○ Much higher (1)
- ○ Somewhat higher (2)
- ○ No difference (3)
- ○ Somewhat lower (4)
- ○ Much lower (5)

Page Break

27
For numeric variables, median (minimum, maximum) is reported. For categorical variables, mode (all values present) is reported.

ETT:
Hours intubated
Subgroup: 664 (388, 3064)
Population: 132 (12, 3072)

Ventilator:
Respiratory rate (set)
Subgroup: 16 (0, 27.5)
Population: 20 (0, 60)

Other Clinical Data:
Glasgow coma scale score: 8-12 hours ago
Subgroup: 9 (5, 15)
Population: 8 (3, 15)

Do patients in the subgroup have a higher or lower chance of **successful extubation** 12 hours from now compared with patients in the population?

○ Much higher (1)

○ Somewhat higher (2)

○ No difference (3)

○ Somewhat lower (4)

○ Much lower (5)

Page Break

28
For numeric variables, median (minimum, maximum) is reported. For categorical variables, mode (all values present) is reported.

Ventilator:
Respiratory rate (set)
Subgroup: 32 (27.7, 60)
Population: 20 (0, 60)

Do patients in the subgroup have a higher or lower chance of **successful extubation** 12 hours from now compared with patients in the population?

○ Much higher (1)

○ Somewhat higher (2)

○ No difference (3)

○ Somewhat lower (4)

○ Much lower (5)

Page Break

29
For numeric variables, median (minimum, maximum) is reported. For categorical variables, mode (all values present) is reported.

ETT:
ETT placed day of surgery
Subgroup: No (No)
Population: No (No, Yes)

Ventilator:
Mean airway pressure (measured)
Subgroup: 10 (3, 14.4)
Population: 11 (3, 35.5)

Other Clinical Data:
State behavioral scale: 8-12 hours ago
Subgroup: -2.5 (-3, -1.8)
Population: -1 (-3, 2)

Do patients in the subgroup have a higher or lower chance of **successful extubation** 12 hours from now compared with patients in the population?

○ Much higher (1)

○ Somewhat higher (2)

○ No difference (3)

○ Somewhat lower (4)

○ Much lower (5)

Page Break

30
For numeric variables, median (minimum, maximum) is reported. For categorical variables, mode (all values present) is reported.

ETT:
ETT placed day of surgery
Subgroup: Yes (Yes)
Population: No (No, Yes)

Ventilator:
Mean airway pressure (measured)
Subgroup: 10 (3, 14.4)
Population: 11 (3, 35.5)

Do patients in the subgroup have a higher or lower chance of **successful extubation** 12 hours from now compared with patients in the population?

- ○ Much higher (1)
- ○ Somewhat higher (2)
- ○ No difference (3)
- ○ Somewhat lower (4)
- ○ Much lower (5)

Page Break

31

For numeric variables, median (minimum, maximum) is reported. For categorical variables, mode (all values present) is reported.

Vital Signs:

Diastolic blood pressure percentile (age-adjusted): Baseline

Subgroup: 0.9 (0.3, 1)

Population: 0.9 (0, 1)

Ventilator:

PEEP (set)

Subgroup: 6 (5, 8.5)

Population: 7 (5, 19)

Other Clinical Data:

Average upper extremity motor response (0 = no movement, 5 = full power): 4-8 hours ago

Subgroup: 2 (0, 3.1)

Population: 2.2 (0, 5)

Do patients in the subgroup have a higher or lower chance of **successful extubation** 12 hours from now compared with patients in the population?

- ○ Much higher (1)
- ○ Somewhat higher (2)
- ○ No difference (3)
- ○ Somewhat lower (4)
- ○ Much lower (5)

Page Break

32
For numeric variables, median (minimum, maximum) is reported. For categorical variables, mode (all values present) is reported.

Ventilator:
FiO2: 4-8 hours ago
Subgroup: 60 (48.1, 100)
Population: 40 (21, 100)

Do patients in the subgroup have a higher or lower chance of **successful extubation** 12 hours from now compared with patients in the population?

○ Much higher (1)

○ Somewhat higher (2)

○ No difference (3)

○ Somewhat lower (4)

○ Much lower (5)

Page Break

33
For numeric variables, median (minimum, maximum) is reported. For categorical variables, mode (all values present) is reported.

ETT:
Spontaneous breathing trial
Subgroup: Ineligible (Ineligible)
Population: Not assessed (Not assessed, Ineligible, Eligible but inconclusive, Failed, Passed)

Ventilator:
PEEP (set): 8-12 hours ago
Subgroup: 7 (5, 9)
Population: 7 (5, 19)

Do patients in the subgroup have a higher or lower chance of **successful extubation** 12 hours from now compared with patients in the population?

- ○ Much higher (1)
- ○ Somewhat higher (2)
- ○ No difference (3)
- ○ Somewhat lower (4)
- ○ Much lower (5)

Page Break

34

For numeric variables, median (minimum, maximum) is reported. For categorical variables, mode (all values present) is reported.

ETT:

Spontaneous breathing trial

Subgroup: Not assessed (Not assessed, Eligible but inconclusive, Failed, Passed)

Population: Not assessed (Not assessed, Ineligible, Eligible but inconclusive, Failed, Passed)

Ventilator:

PEEP (set): 8-12 hours ago

Subgroup: 6 (5, 9)

Population: 7 (5, 19)

Other Clinical Data:

Average lower extremity motor response (0 = no movement, 5 = full power)

Subgroup: 0 (0, 0.4)

Population: 2 (0, 5)

Do patients in the subgroup have a higher or lower chance of **successful extubation** 12 hours from now compared with patients in the population?

- ○ Much higher (1)
- ○ Somewhat higher (2)
- ○ No difference (3)
- ○ Somewhat lower (4)
- ○ Much lower (5)

Page Break

35

For numeric variables, median (minimum, maximum) is reported. For categorical variables, mode (all values present) is reported.

Ventilator:

PEEP (set): 8-12 hours ago

Subgroup: 10 (9.2, 19)

Population: 7 (5, 19)

Do patients in the subgroup have a higher or lower chance of **successful extubation** 12 hours from now compared with patients in the population?

- ○ Much higher (1)
- ○ Somewhat higher (2)
- ○ No difference (3)
- ○ Somewhat lower (4)
- ○ Much lower (5)

Page Break

36

For numeric variables, median (minimum, maximum) is reported. For categorical variables, mode (all values present) is reported.

Vital Signs:

Pulse percentile (age-adjusted): 4-8 hours ago

Subgroup: 41.2 (0, 69.1)

Population: 69.8 (0, 100)

Ventilator:

Respiratory rate (set)

Subgroup: 16 (0, 22.5)

Population: 20 (0, 60)

Do patients in the subgroup have a higher or lower chance of **successful extubation** 12 hours from now compared with patients in the population?

- Much higher (1)
- Somewhat higher (2)
- No difference (3)
- Somewhat lower (4)
- Much lower (5)

Page Break

37
For numeric variables, median (minimum, maximum) is reported. For categorical variables, mode (all values present) is reported.

Ventilator:
Respiratory rate (set)
Subgroup: 30 (22.7, 60)
Population: 20 (0, 60)

Do patients in the subgroup have a higher or lower chance of **successful extubation** 12 hours from now compared with patients in the population?

- ○ Much higher (1)
- ○ Somewhat higher (2)
- ○ No difference (3)
- ○ Somewhat lower (4)
- ○ Much lower (5)

Page Break

38
For numeric variables, median (minimum, maximum) is reported. For categorical variables, mode (all values present) is reported.

Ventilator:
Peak inspiratory pressure (measured)
Subgroup: 16 (6, 19.8)
Population: 21.2 (5.7, 56)

Other Clinical Data:
Intake/output total ml per kg over the last 72 hours
Subgroup: 7.6 (-348.9, 43.5)
Population: 34.7 (-436.3, 500.2)

Do patients in the subgroup have a higher or lower chance of **successful extubation** 12 hours from now compared with patients in the population?

○ Much higher (1)

○ Somewhat higher (2)

○ No difference (3)

○ Somewhat lower (4)

○ Much lower (5)

Page Break

39
For numeric variables, median (minimum, maximum) is reported. For categorical variables, mode (all values present) is reported.

Other Clinical Data:
Intake/output total ml per kg over the last 72 hours
Subgroup: 88.6 (43.5, 500.2)
Population: 34.7 (-436.3, 500.2)

Do patients in the subgroup have a higher or lower chance of **successful extubation** 12 hours from now compared with patients in the population?

○ Much higher (1)

○ Somewhat higher (2)

○ No difference (3)

○ Somewhat lower (4)

○ Much lower (5)

Page Break

40
For numeric variables, median (minimum, maximum) is reported. For categorical variables, mode (all values present) is reported.

ETT:
ETT placed day of surgery
Subgroup: No (No)
Population: No (No, Yes)

Ventilator:
Respiratory rate (set)
Subgroup: 16 (2, 20)
Population: 20 (0, 60)

PEEP (set)
Subgroup: 8 (6.7, 19)
Population: 7 (5, 19)

Do patients in the subgroup have a higher or lower chance of **successful extubation** 12 hours from now compared with patients in the population?

○ Much higher (1)

○ Somewhat higher (2)

○ No difference (3)

○ Somewhat lower (4)

○ Much lower (5)

Page Break

41

For numeric variables, median (minimum, maximum) is reported. For categorical variables, mode (all values present) is reported.

ETT:

ETT placed day of surgery

Subgroup: No (No)

Population: No (No, Yes)

Ventilator:

Respiratory rate (set)

Subgroup: 28 (20.3, 60)

Population: 20 (0, 60)

Do patients in the subgroup have a higher or lower chance of **successful extubation** 12 hours from now compared with patients in the population?

○ Much higher (1)

○ Somewhat higher (2)

○ No difference (3)

○ Somewhat lower (4)

○ Much lower (5)

Page Break

42
For numeric variables, median (minimum, maximum) is reported. For categorical variables, mode (all values present) is reported.

ETT:
ETT placed day of surgery
Subgroup: Yes (Yes)
Population: No (No, Yes)

Do patients in the subgroup have a higher or lower chance of **successful extubation** 12 hours from now compared with patients in the population?

- ○ Much higher (1)
- ○ Somewhat higher (2)
- ○ No difference (3)
- ○ Somewhat lower (4)
- ○ Much lower (5)

Page Break

43
For numeric variables, median (minimum, maximum) is reported. For categorical variables, mode (all values present) is reported.

Other Clinical Data:
State behavioral scale
Subgroup: -2.3 (-3, -1.4)
Population: -1 (-3, 2)

Do patients in the subgroup have a higher or lower chance of **successful extubation** 12 hours from now compared with patients in the population?

○ Much higher (1)

○ Somewhat higher (2)

○ No difference (3)

○ Somewhat lower (4)

○ Much lower (5)

Page Break

44

For numeric variables, median (minimum, maximum) is reported. For categorical variables, mode (all values present) is reported.

ETT:

Spontaneous breathing trial: 8-12 hours ago

Subgroup: Ineligible (Ineligible)

Population: Not assessed (Not assessed, Ineligible, Eligible but inconclusive, Failed, Passed)

Other Clinical Data:

State behavioral scale

Subgroup: -0.2 (-1.3, 2)

Population: -1 (-3, 2)

Do patients in the subgroup have a higher or lower chance of **successful extubation** 12 hours from now compared with patients in the population?

- ○ Much higher (1)
- ○ Somewhat higher (2)
- ○ No difference (3)
- ○ Somewhat lower (4)
- ○ Much lower (5)

Page Break

45
For numeric variables, median (minimum, maximum) is reported. For categorical variables, mode (all values present) is reported.

ETT:
Spontaneous breathing trial: 8-12 hours ago
Subgroup: Not assessed (Not assessed, Eligible but inconclusive, Failed, Passed)
Population: Not assessed (Not assessed, Ineligible, Eligible but inconclusive, Failed, Passed)

Hours intubated
Subgroup: 12 (12, 12)
Population: 132 (12, 3072)

Other Clinical Data:
State behavioral scale
Subgroup: -0.3 (-1.3, 2)
Population: -1 (-3, 2)

Do patients in the subgroup have a higher or lower chance of **successful extubation** 12 hours from now compared with patients in the population?

- ○ Much higher (1)
- ○ Somewhat higher (2)
- ○ No difference (3)
- ○ Somewhat lower (4)
- ○ Much lower (5)

Page Break

46

For numeric variables, median (minimum, maximum) is reported. For categorical variables, mode (all values present) is reported.

ETT:

Spontaneous breathing trial: 8-12 hours ago

Subgroup: Not assessed (Not assessed, Eligible but inconclusive, Failed, Passed)

Population: Not assessed (Not assessed, Ineligible, Eligible but inconclusive, Failed, Passed)

Hours intubated

Subgroup: 132 (16, 3072)

Population: 132 (12, 3072)

Other Clinical Data:

State behavioral scale

Subgroup: -0.1 (-1.4, 2)

Population: -1 (-3, 2)

Do patients in the subgroup have a higher or lower chance of **successful extubation** 12 hours from now compared with patients in the population?

- ◯ Much higher (1)
- ◯ Somewhat higher (2)
- ◯ No difference (3)
- ◯ Somewhat lower (4)
- ◯ Much lower (5)

Page Break

End of Block: Imported Block 1 - Feb 7, 2024
